# Supplementary figures and images for: Binding and sequestration of poison frog alkaloids by a plasma globulin
Source: eLife. 2023 Dec 19;12:e85096. doi: 10.7554/eLife.85096 (PMC10783871; doi:10.7554/eLife.85096)

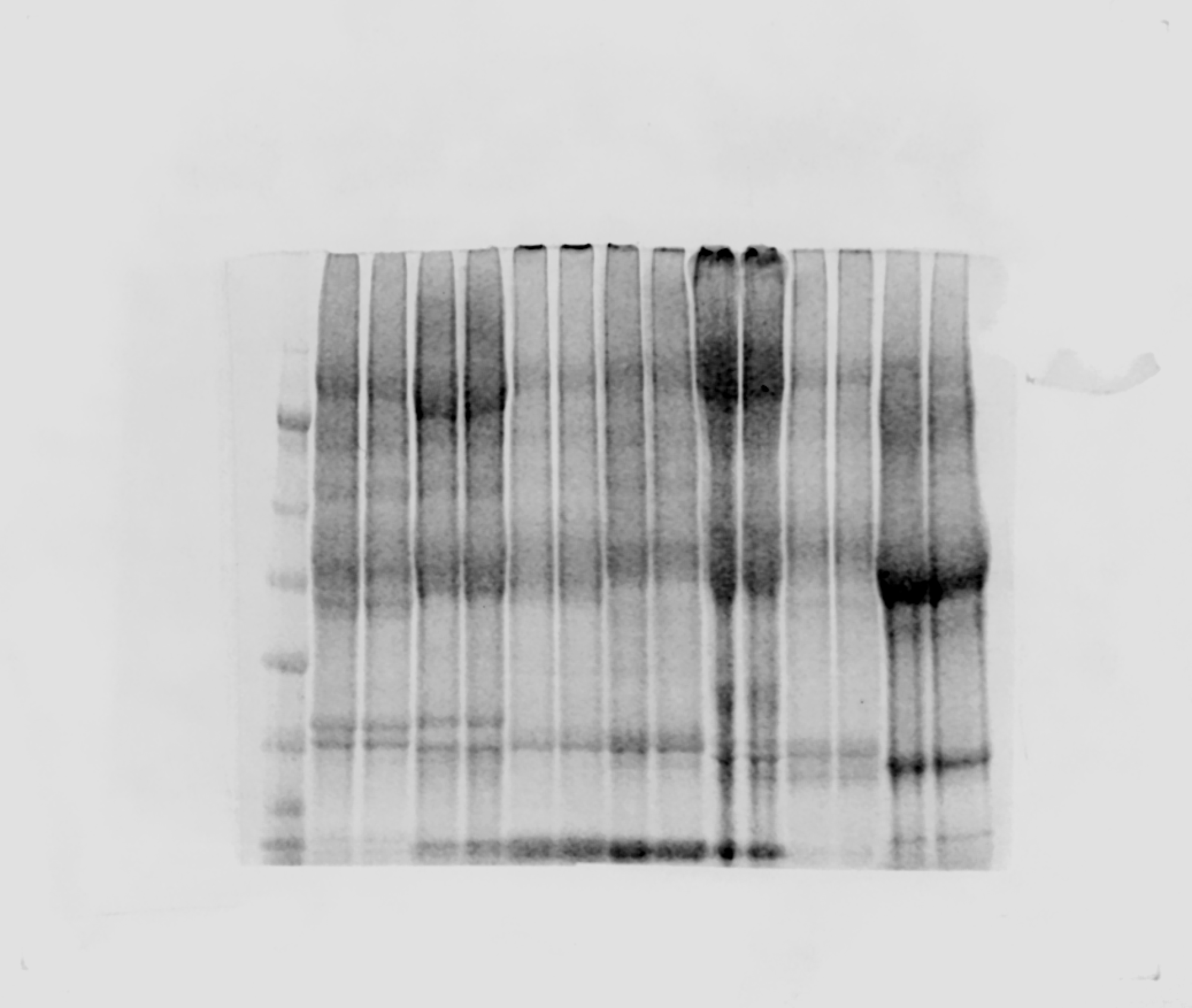

Supplement: Figure 1—source data 1. [file elife-85096-fig1-data1.zip › Figure1_sourcedata/1B_coomassie.tif]

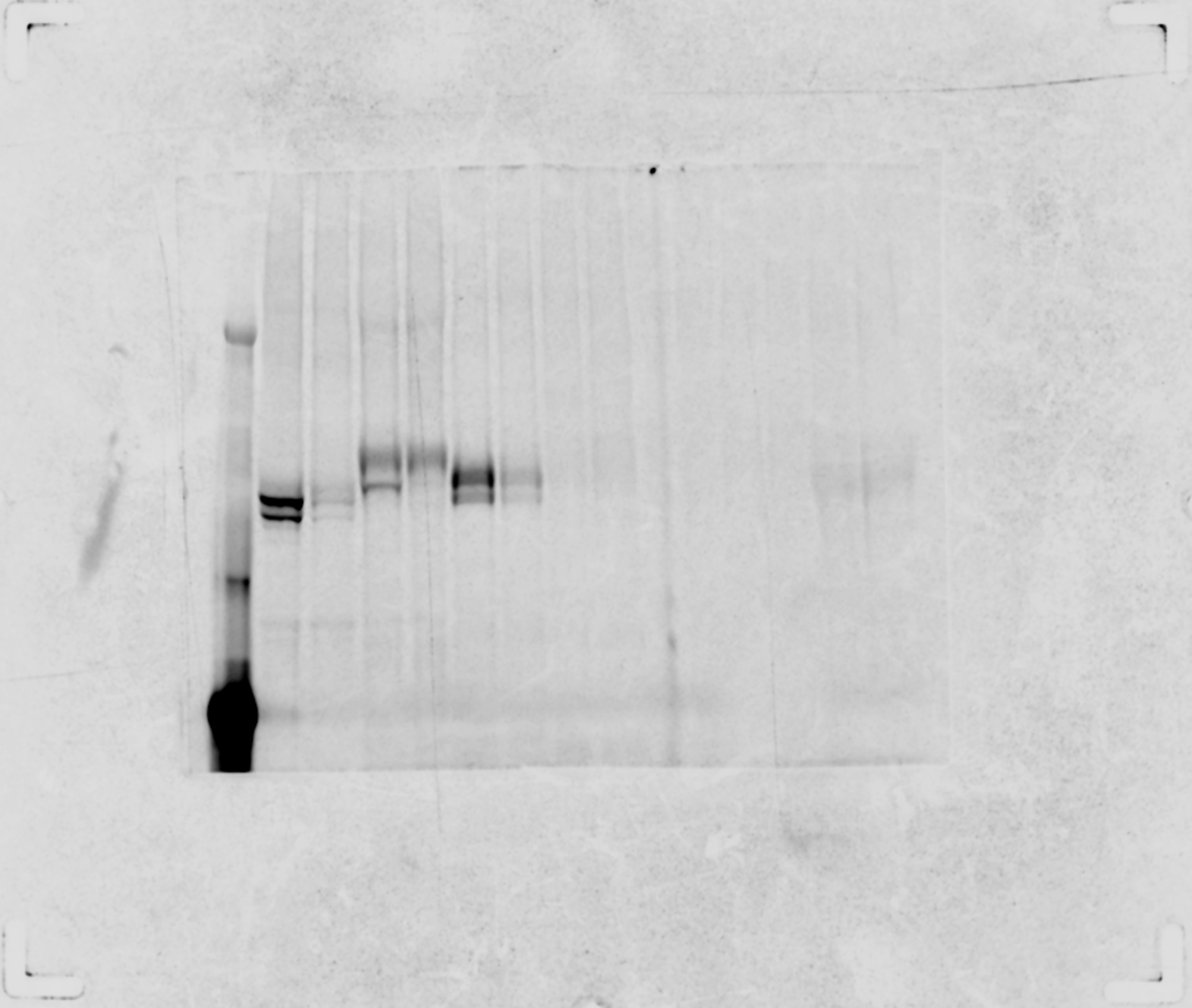

Supplement: Figure 1—source data 1. [file elife-85096-fig1-data1.zip › Figure1_sourcedata/1B_TAMRA.tif]

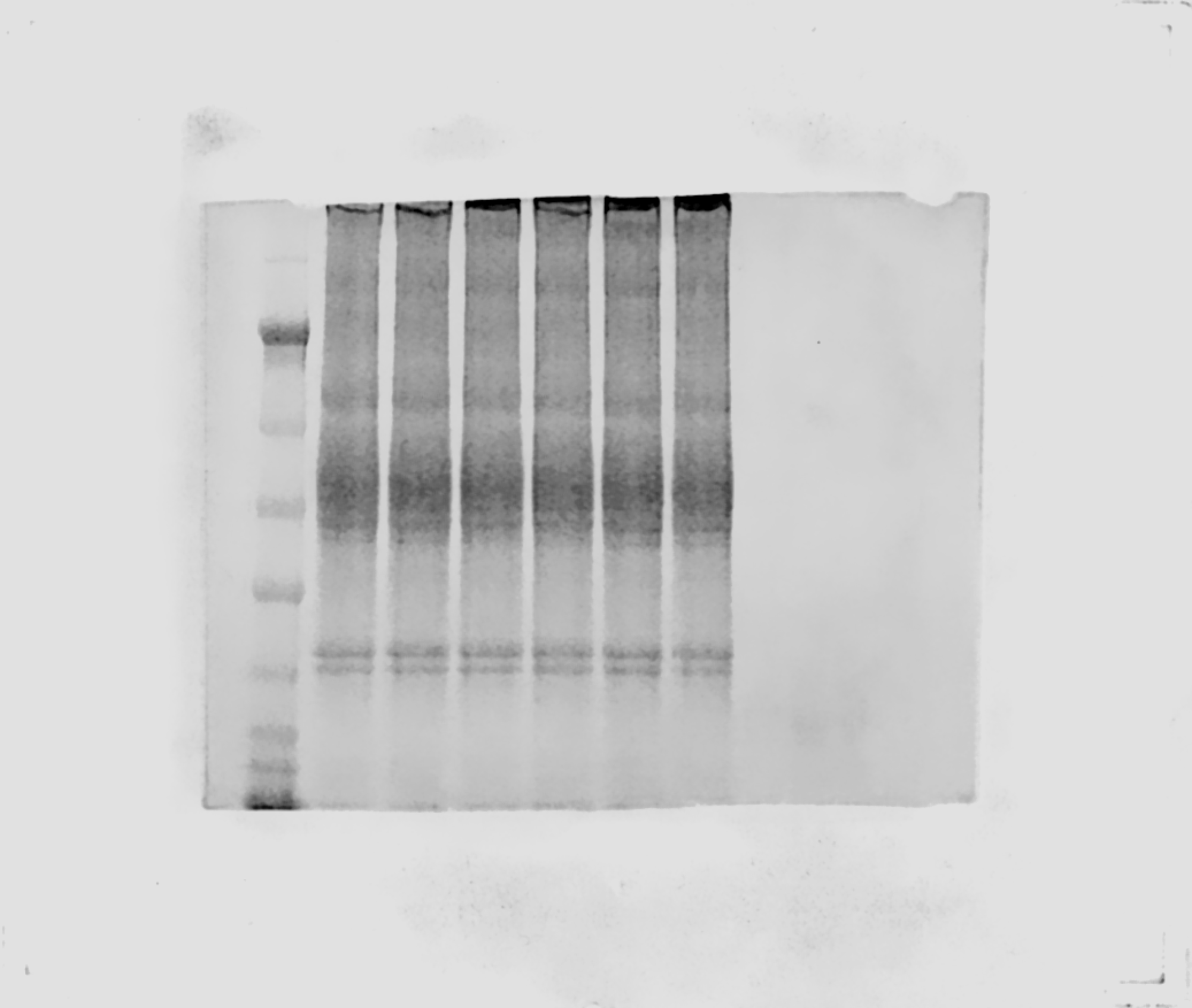

Supplement: Figure 1—source data 1. [file elife-85096-fig1-data1.zip › Figure1_sourcedata/1C_coomassie.tif]

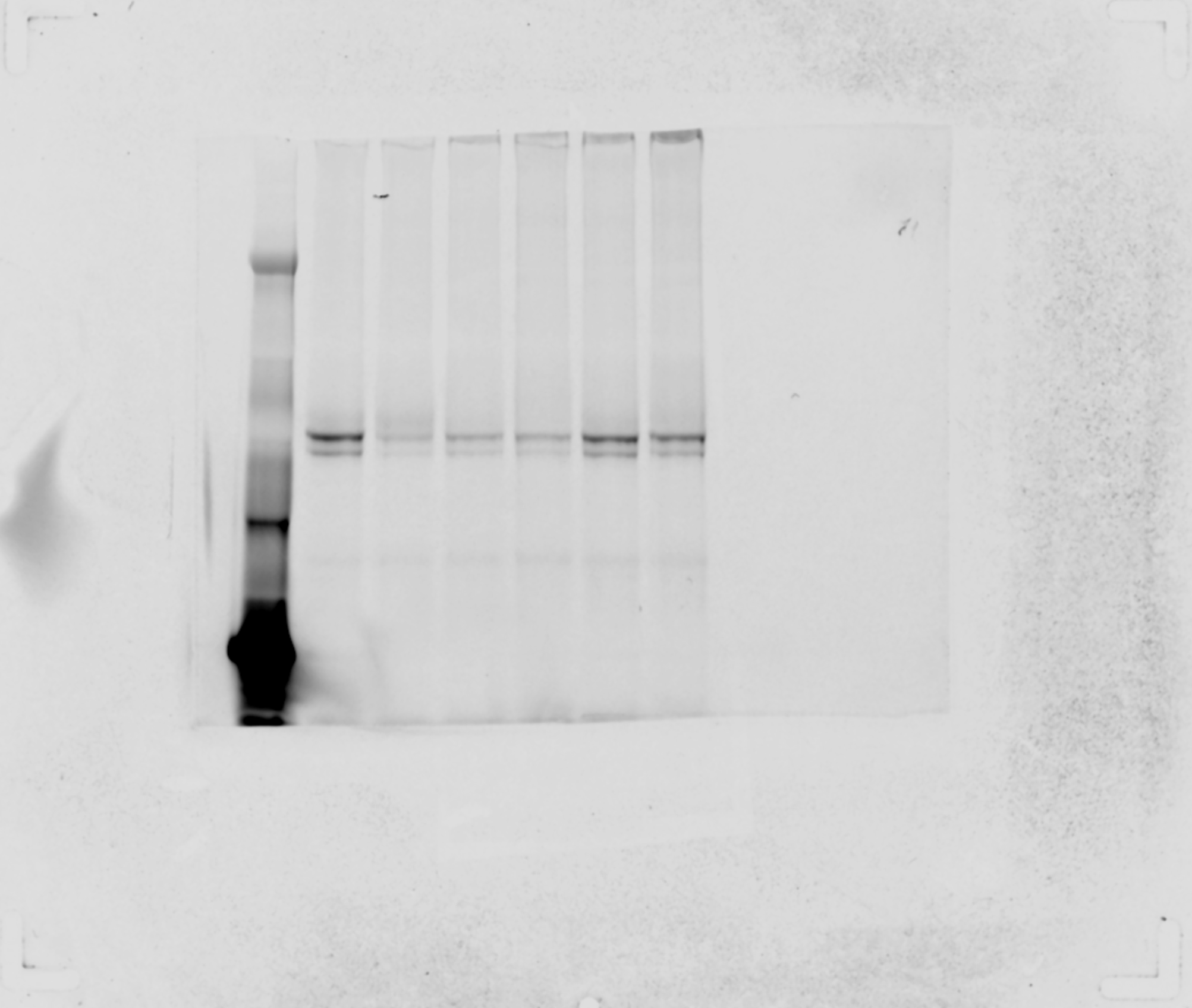

Supplement: Figure 1—source data 1. [file elife-85096-fig1-data1.zip › Figure1_sourcedata/1C_TAMRA.tif]

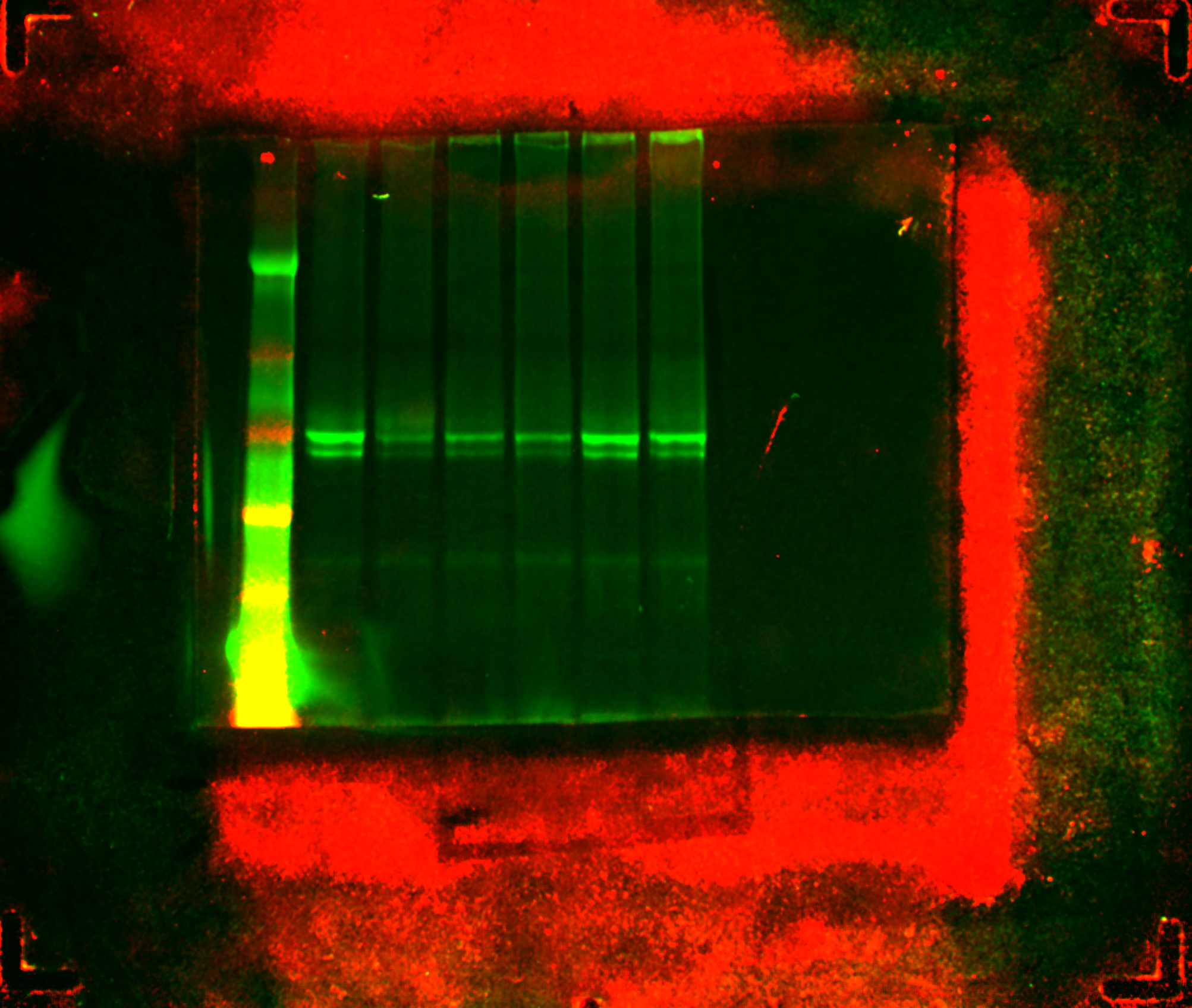

Supplement: Figure 1—source data 1. [file elife-85096-fig1-data1.zip › Figure1_sourcedata/1C_TAMRAladder.tif]

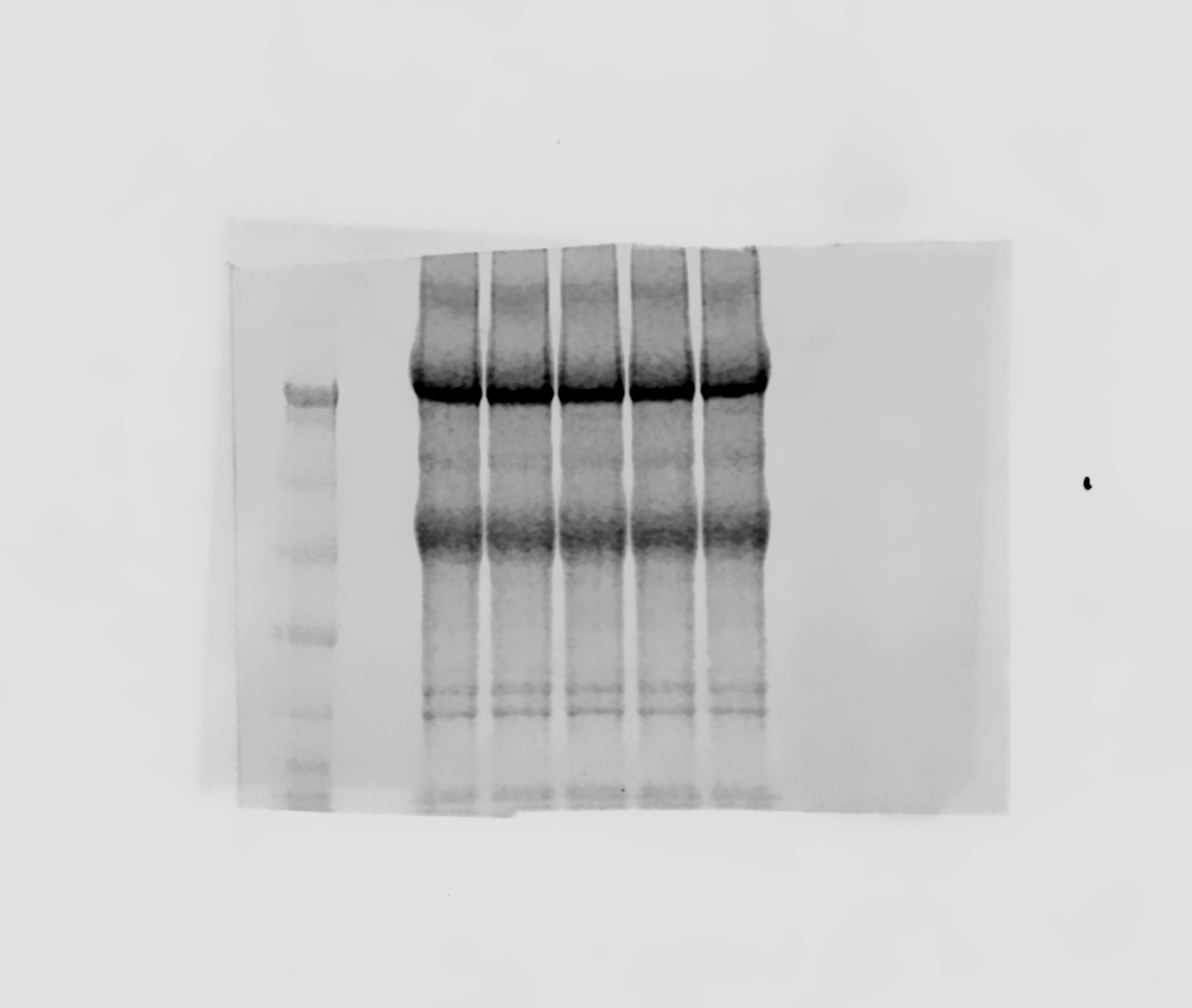

Supplement: Figure 1—source data 1. [file elife-85096-fig1-data1.zip › Figure1_sourcedata/1D_coomassie.tif]

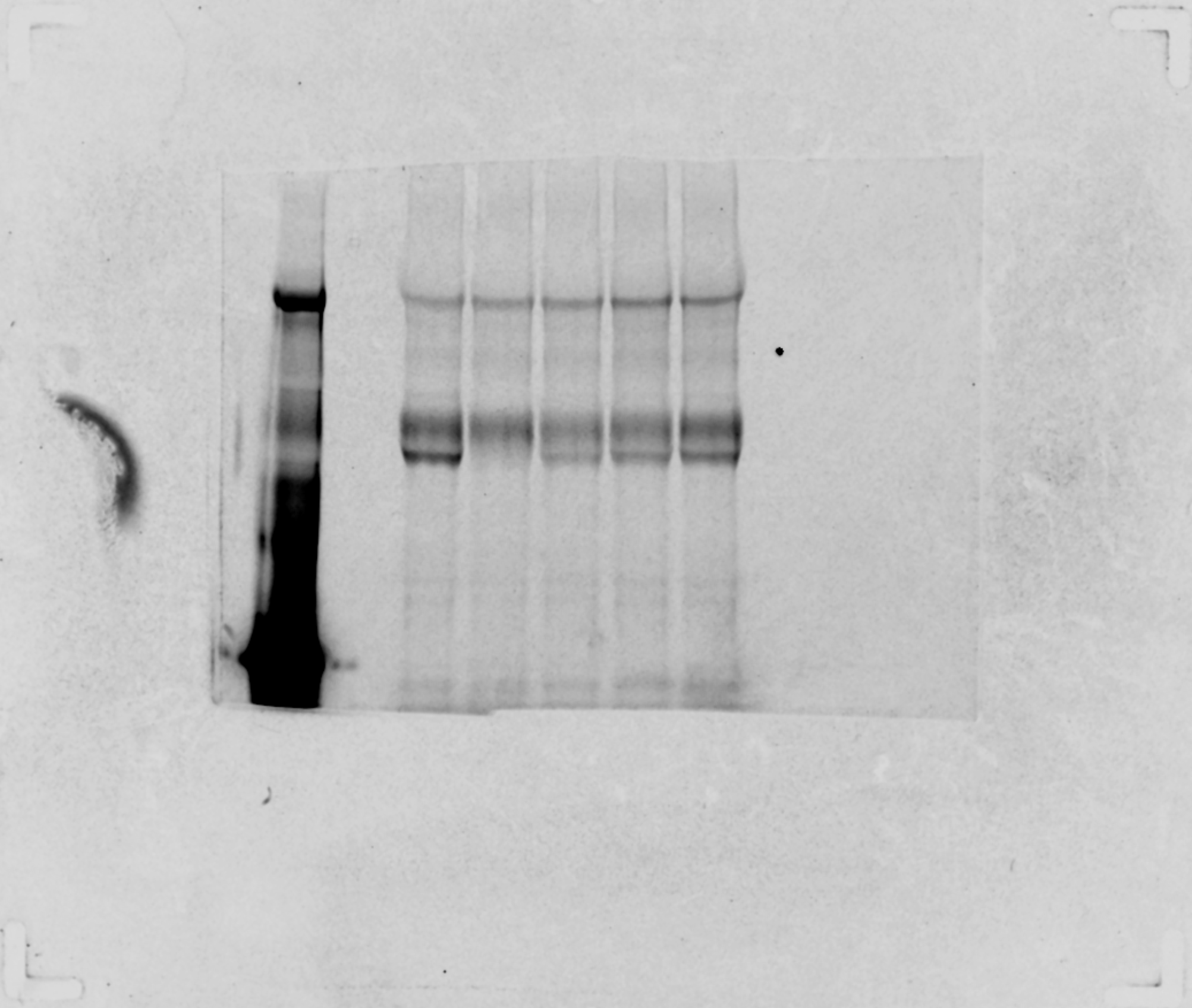

Supplement: Figure 1—source data 1. [file elife-85096-fig1-data1.zip › Figure1_sourcedata/1D_TAMRA.tif]

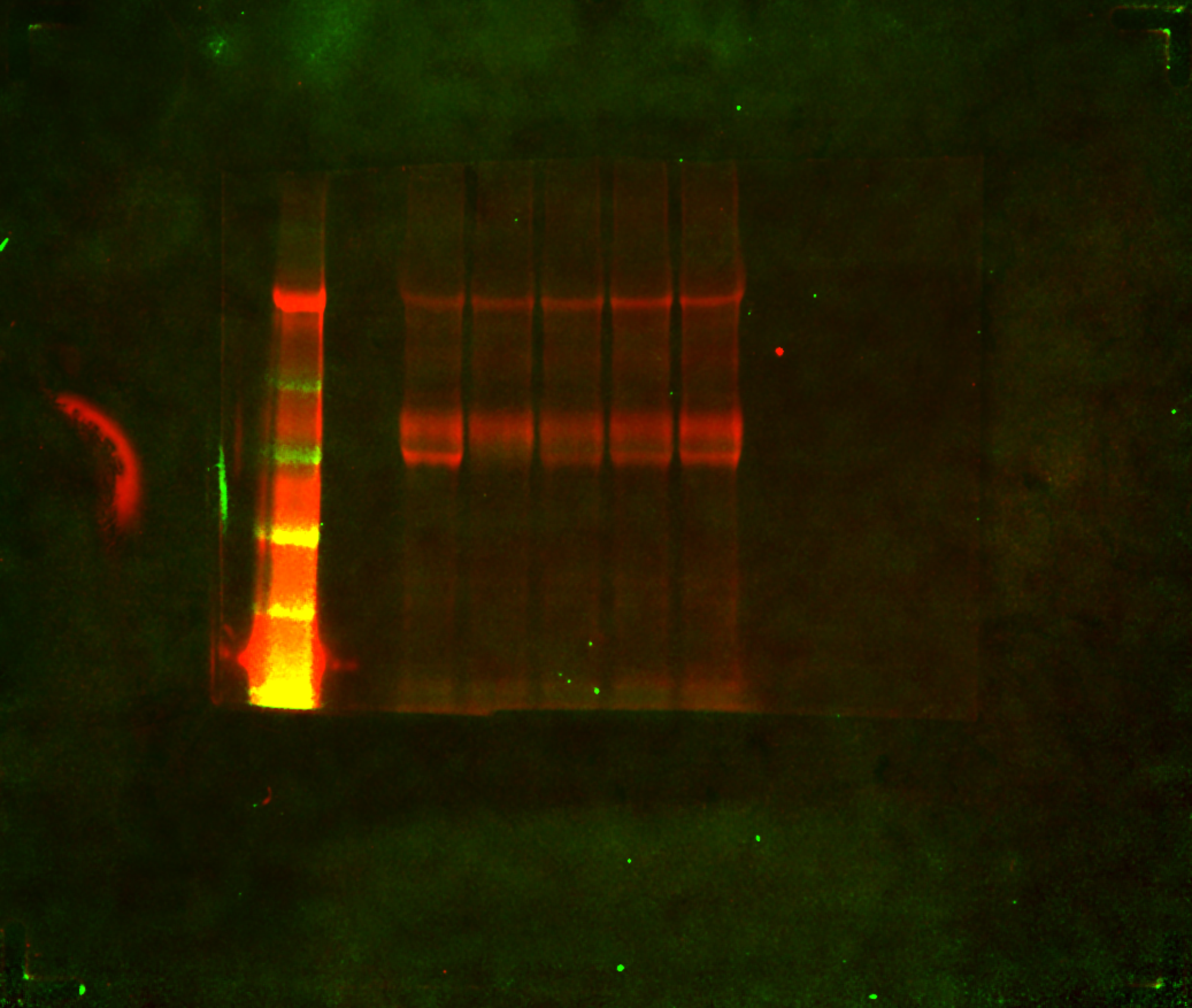

Supplement: Figure 1—source data 1. [file elife-85096-fig1-data1.zip › Figure1_sourcedata/1D_TAMRAladder.tif]

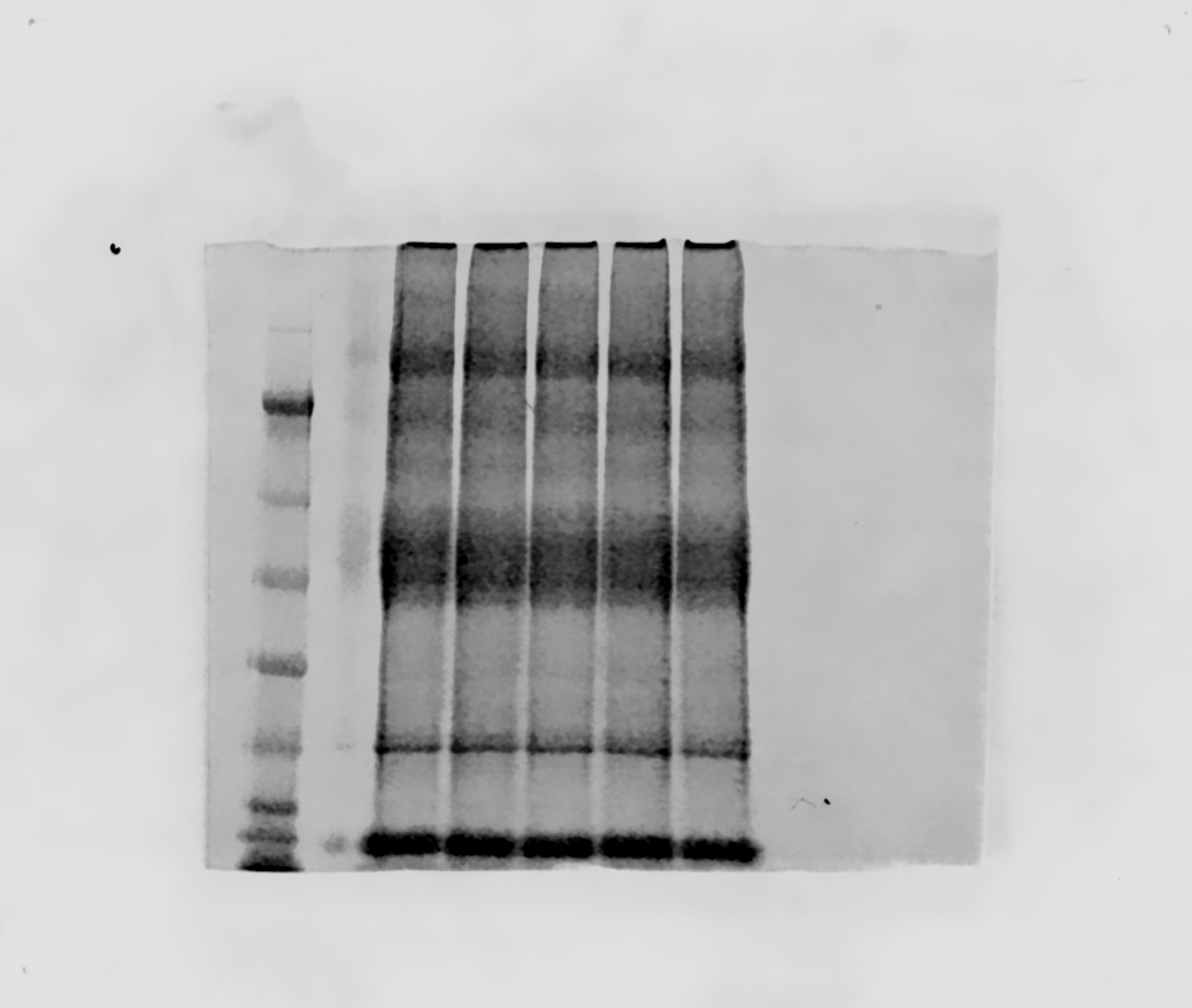

Supplement: Figure 1—source data 1. [file elife-85096-fig1-data1.zip › Figure1_sourcedata/1E_coomassie.tif]

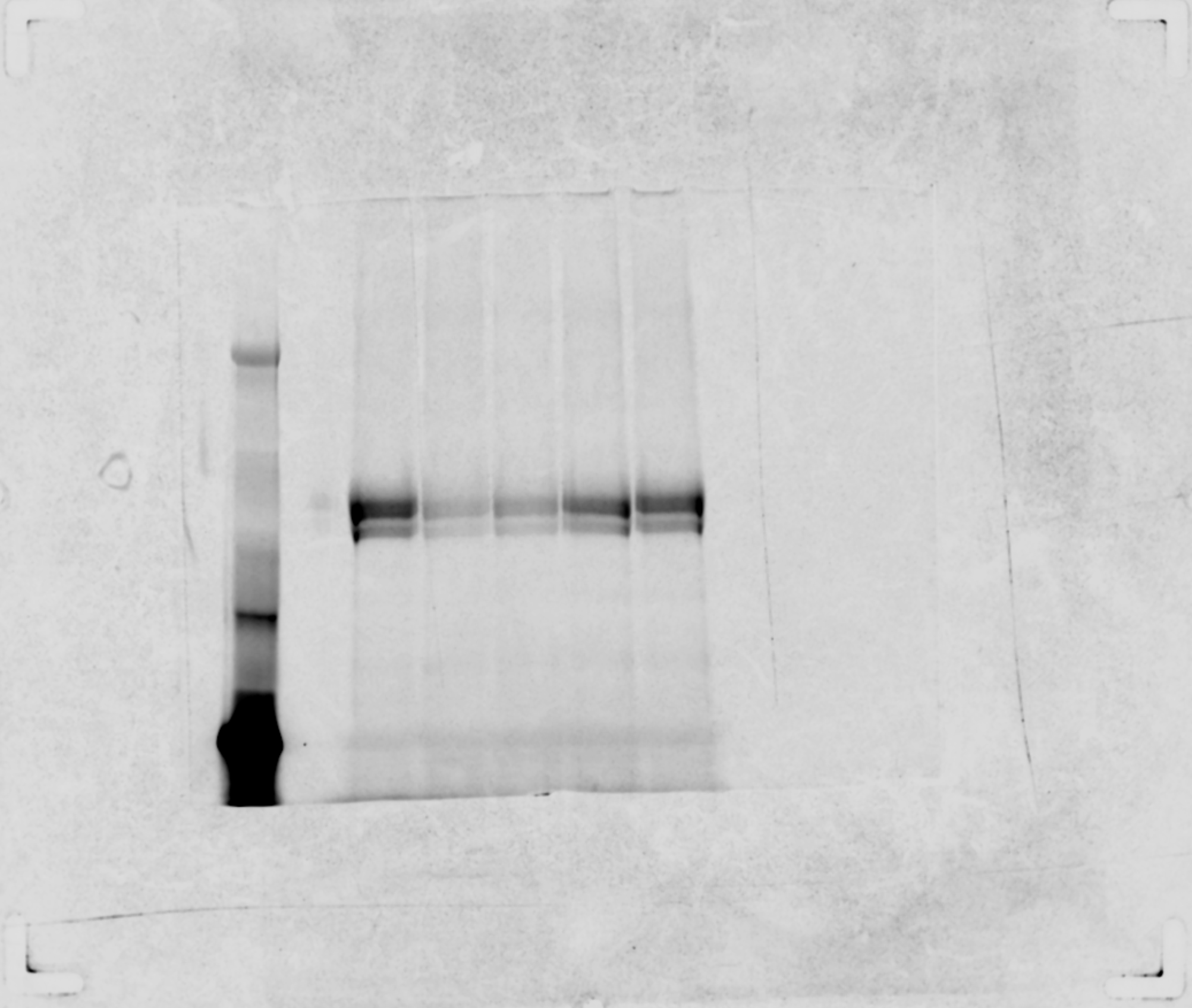

Supplement: Figure 1—source data 1. [file elife-85096-fig1-data1.zip › Figure1_sourcedata/1E_TAMRA.tif]

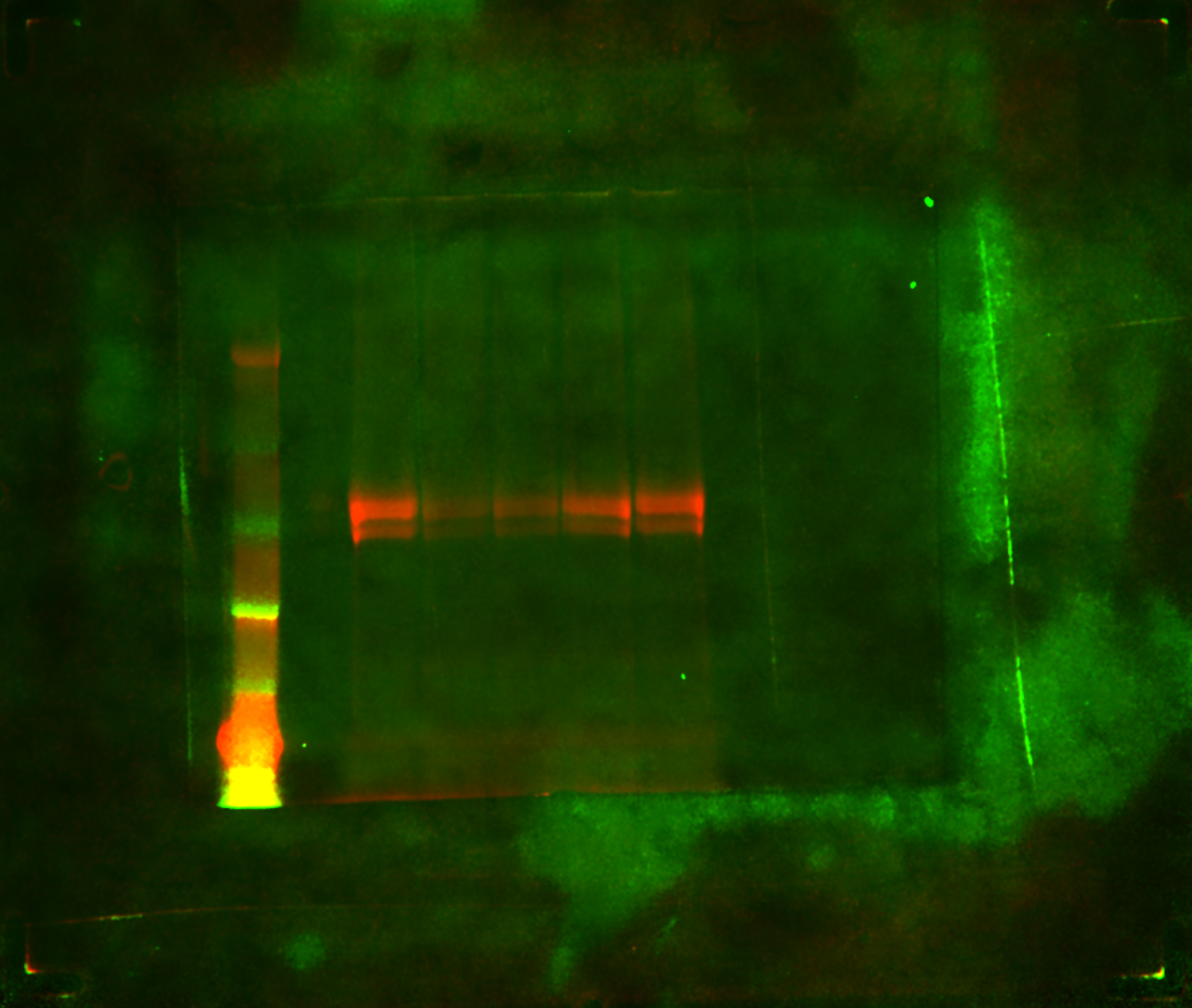

Supplement: Figure 1—source data 1. [file elife-85096-fig1-data1.zip › Figure1_sourcedata/1E_TAMRAladder.tif]

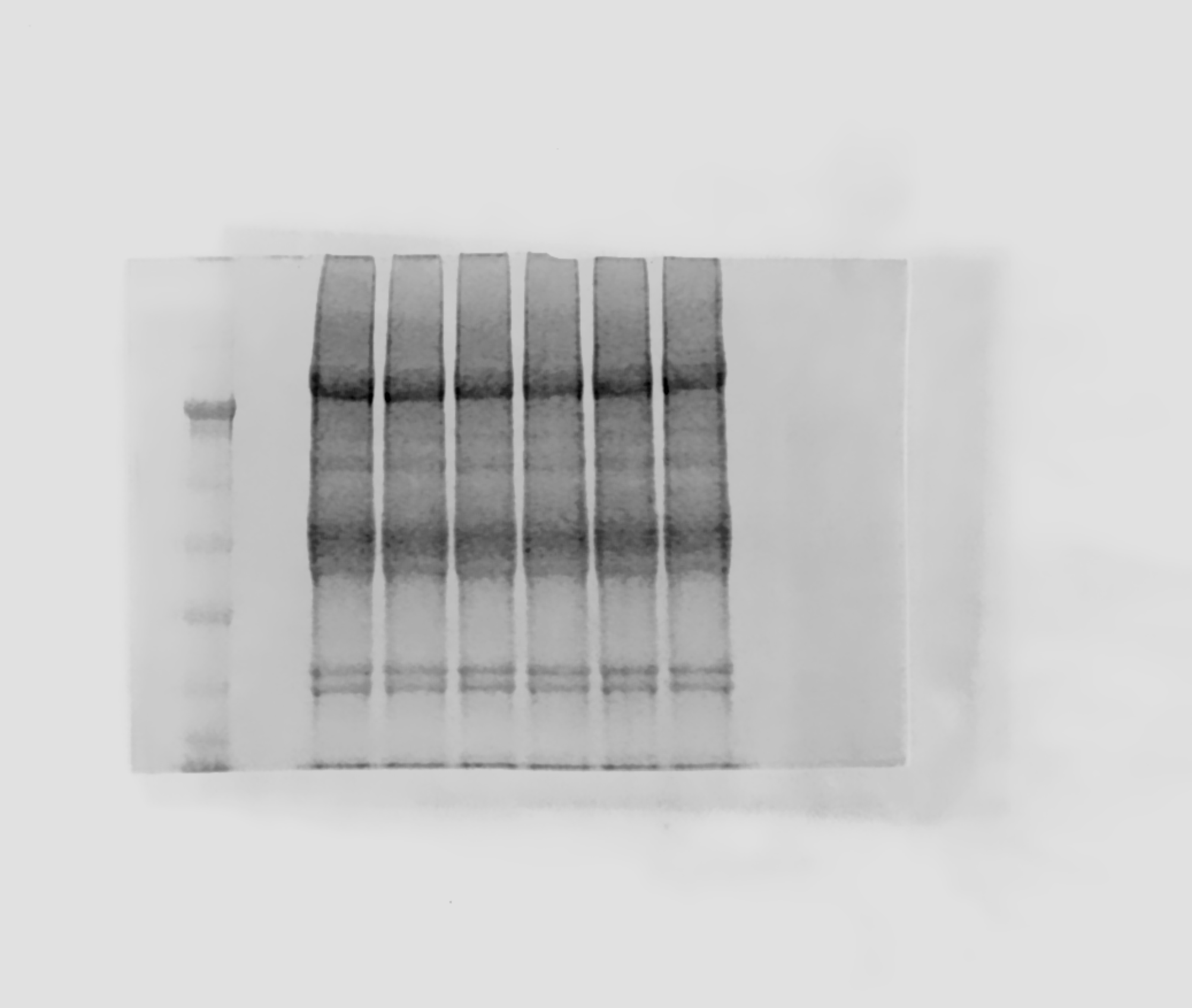

Supplement: Figure 1—figure supplement 1—source data 1. [file elife-85096-fig1-figsupp1-data1.zip › Figure1-fs1-sd1/S1A_coomassie.tif]

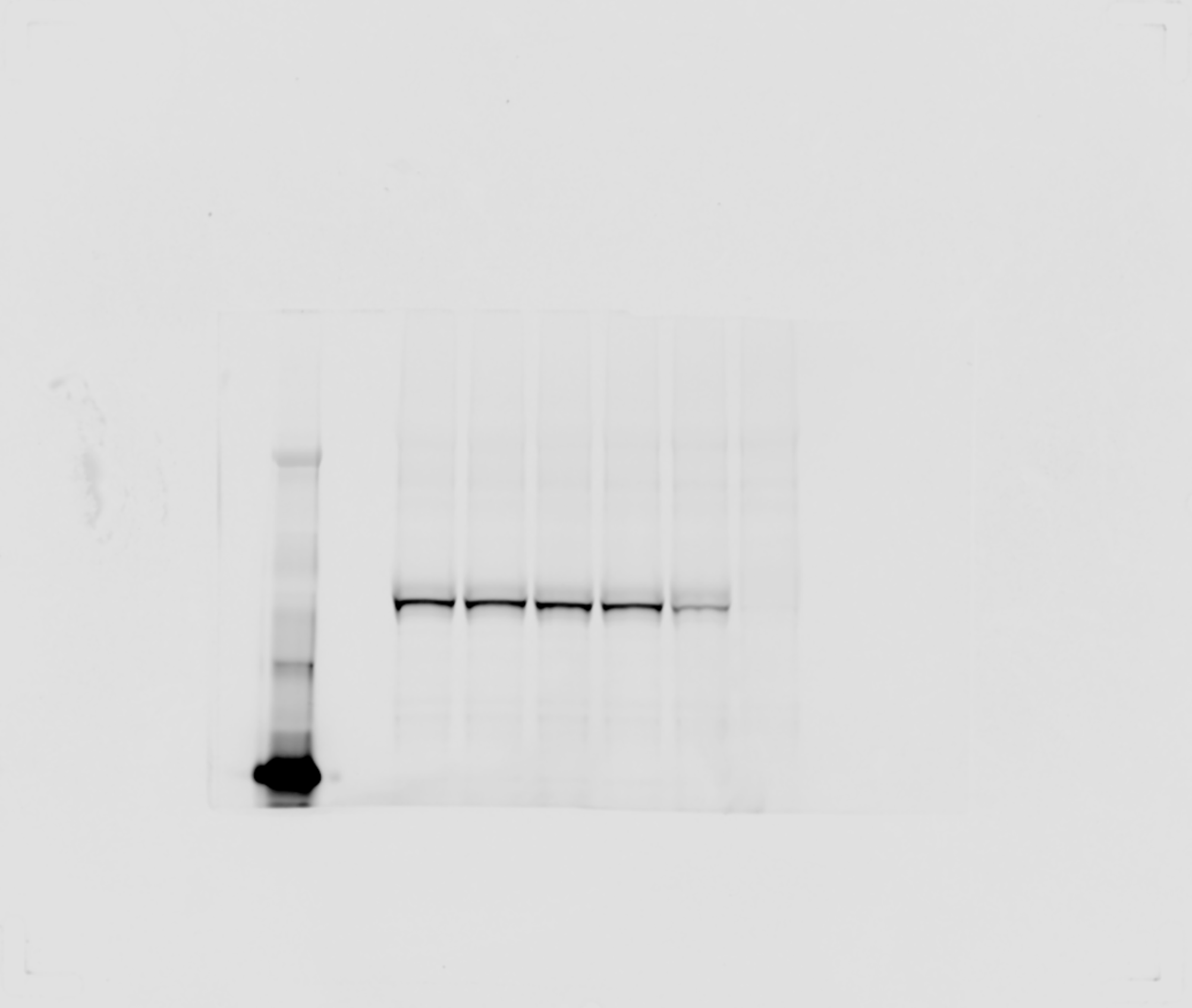

Supplement: Figure 1—figure supplement 1—source data 1. [file elife-85096-fig1-figsupp1-data1.zip › Figure1-fs1-sd1/S1A_TAMRA.tif]

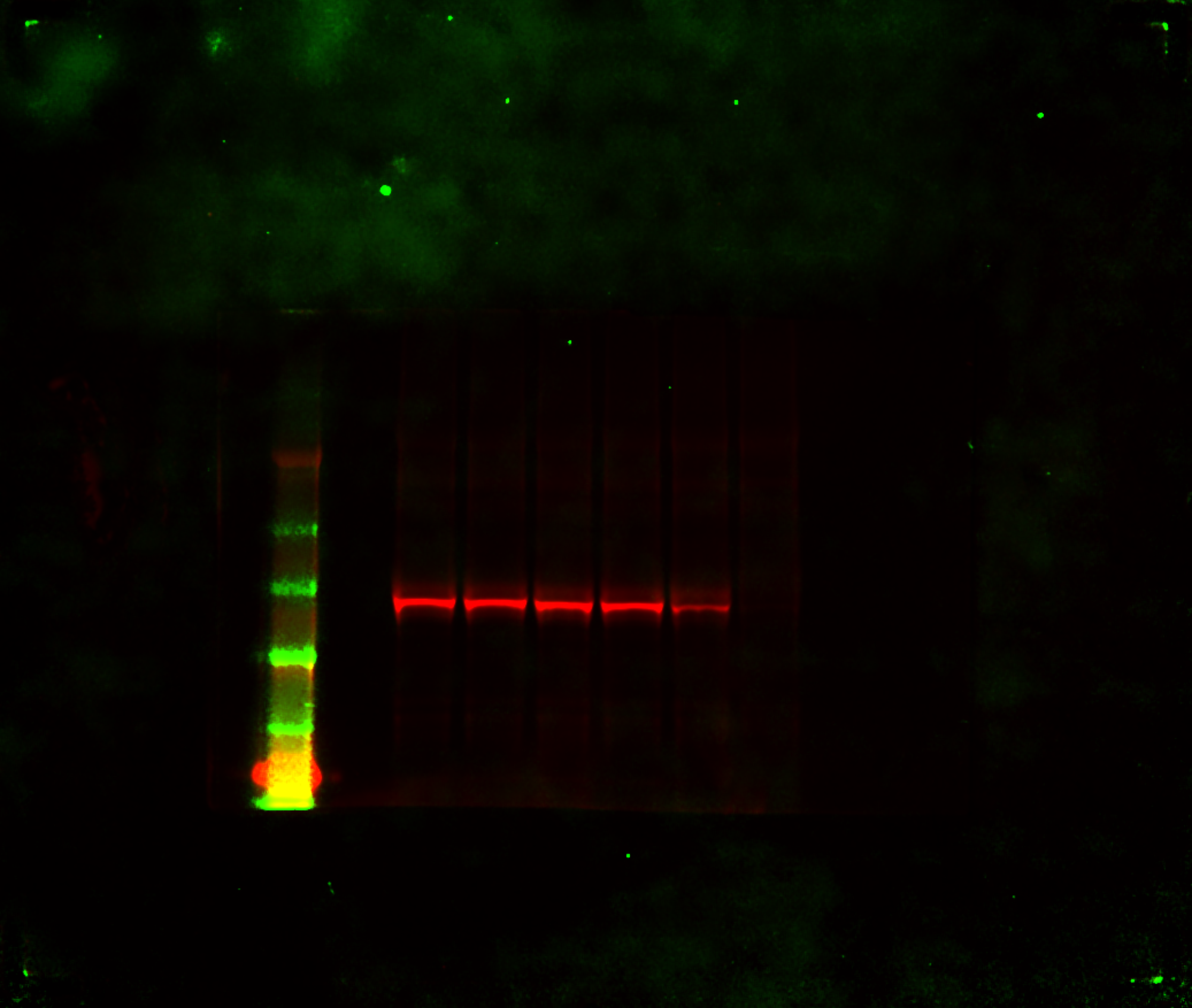

Supplement: Figure 1—figure supplement 1—source data 1. [file elife-85096-fig1-figsupp1-data1.zip › Figure1-fs1-sd1/S1A_TAMRAladder.tif]

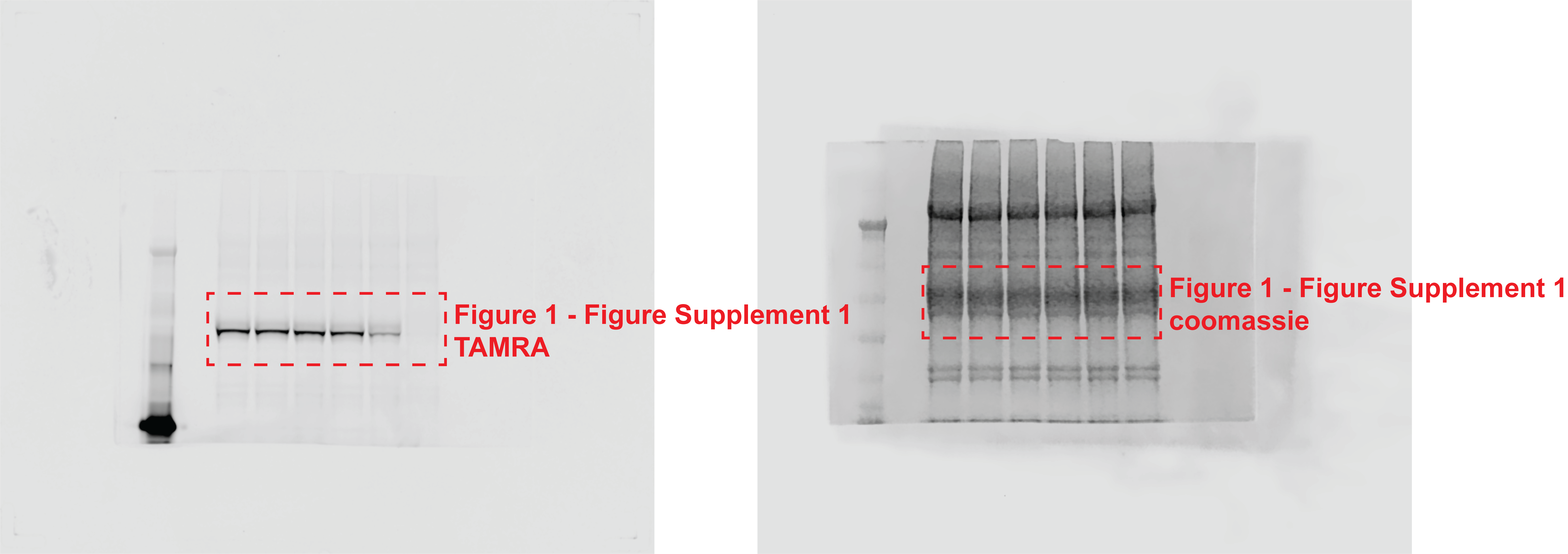

Supplement: Figure 1—figure supplement 1—source data 1. [file elife-85096-fig1-figsupp1-data1.zip › Figure1-fs1-sd1/Figure1_fs1_gelCroppingBounds.png]

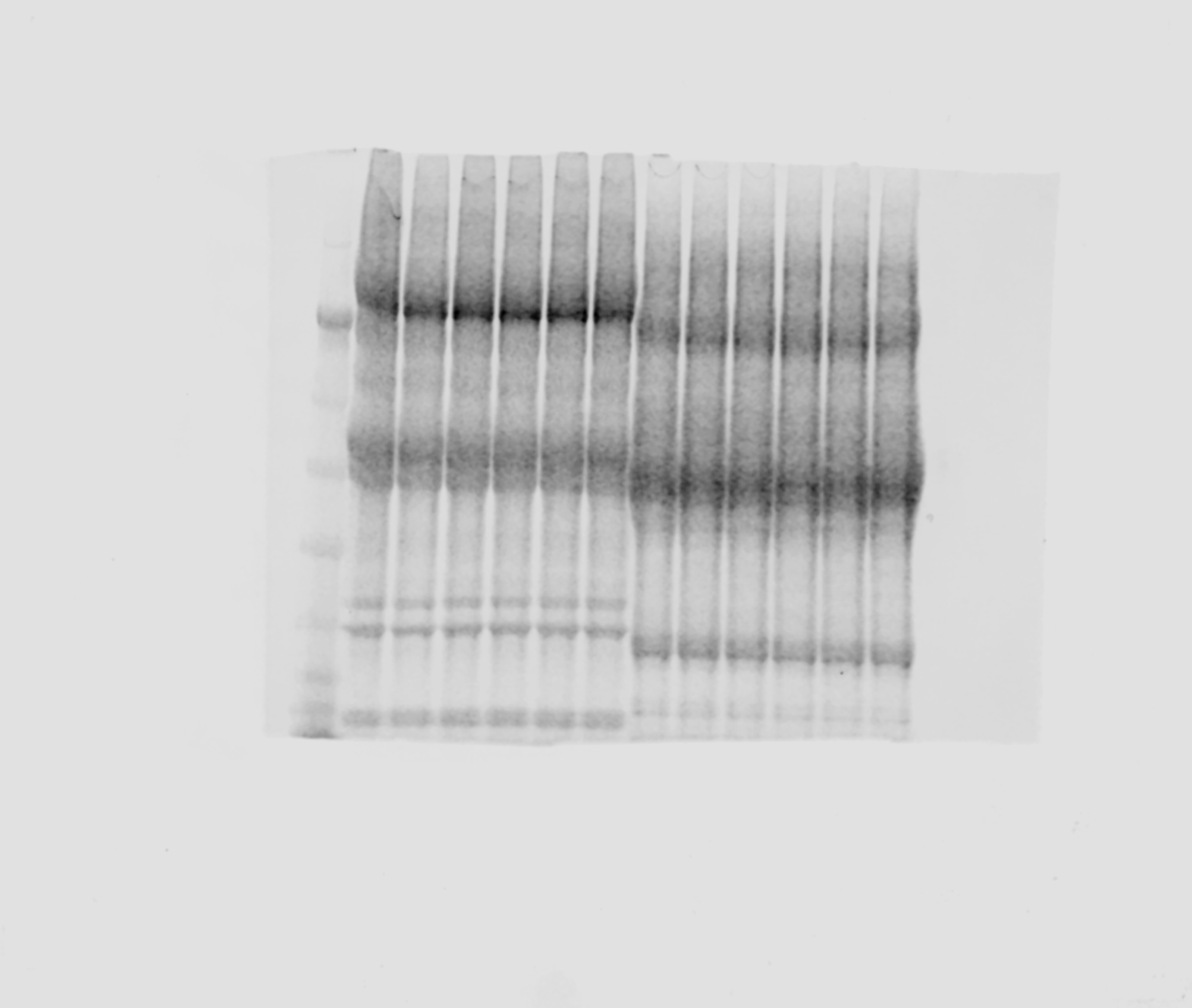

Supplement: Figure 1—figure supplement 2—source data 1. [file elife-85096-fig1-figsupp2-data1.zip › Figure1-fs2-sd1/S1B_first6wells_coomassie.tif]

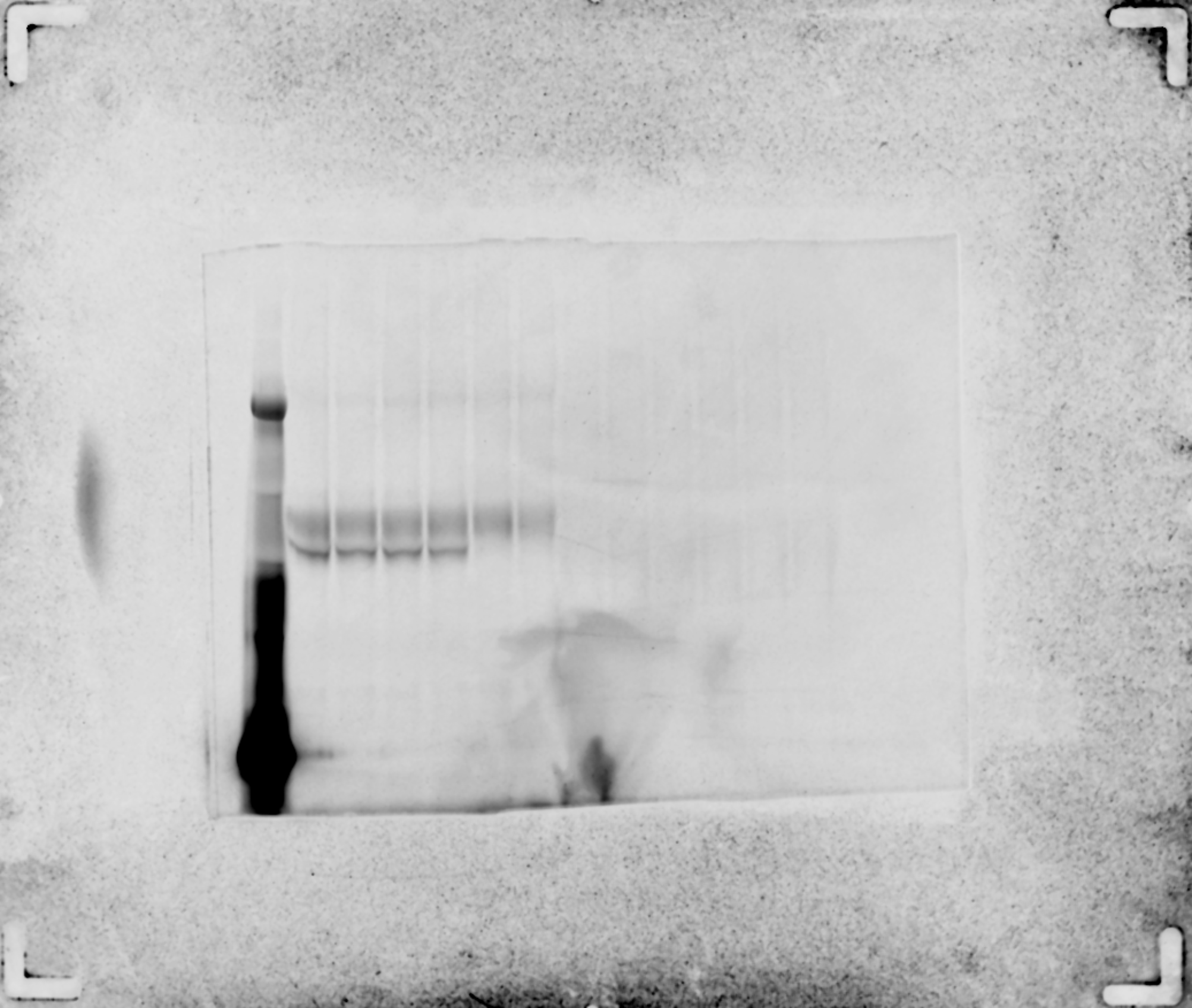

Supplement: Figure 1—figure supplement 2—source data 1. [file elife-85096-fig1-figsupp2-data1.zip › Figure1-fs2-sd1/S1B_first6wells_TAMRA.tif]

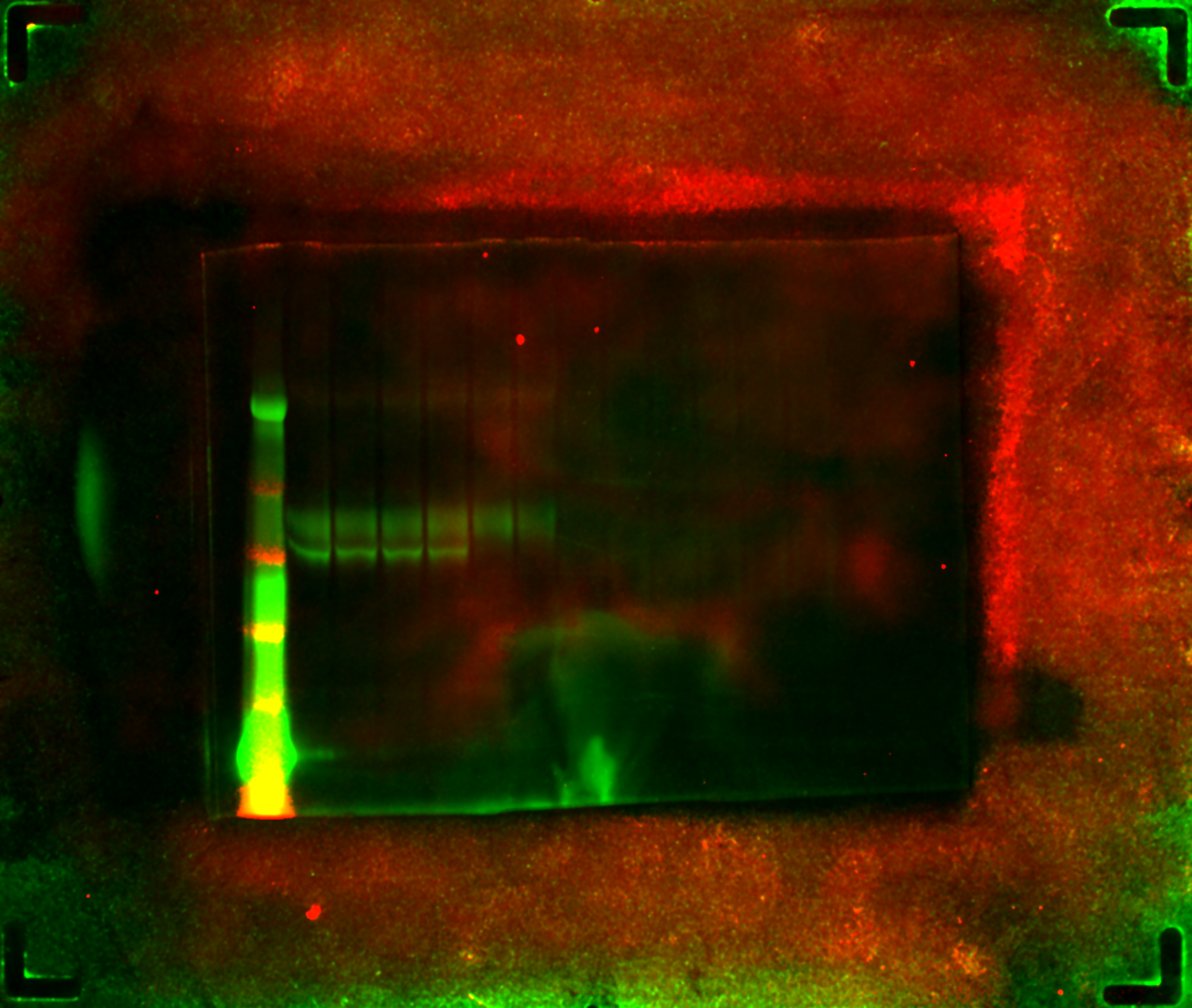

Supplement: Figure 1—figure supplement 2—source data 1. [file elife-85096-fig1-figsupp2-data1.zip › Figure1-fs2-sd1/S1B_first6wells_TAMRAladder.tif]

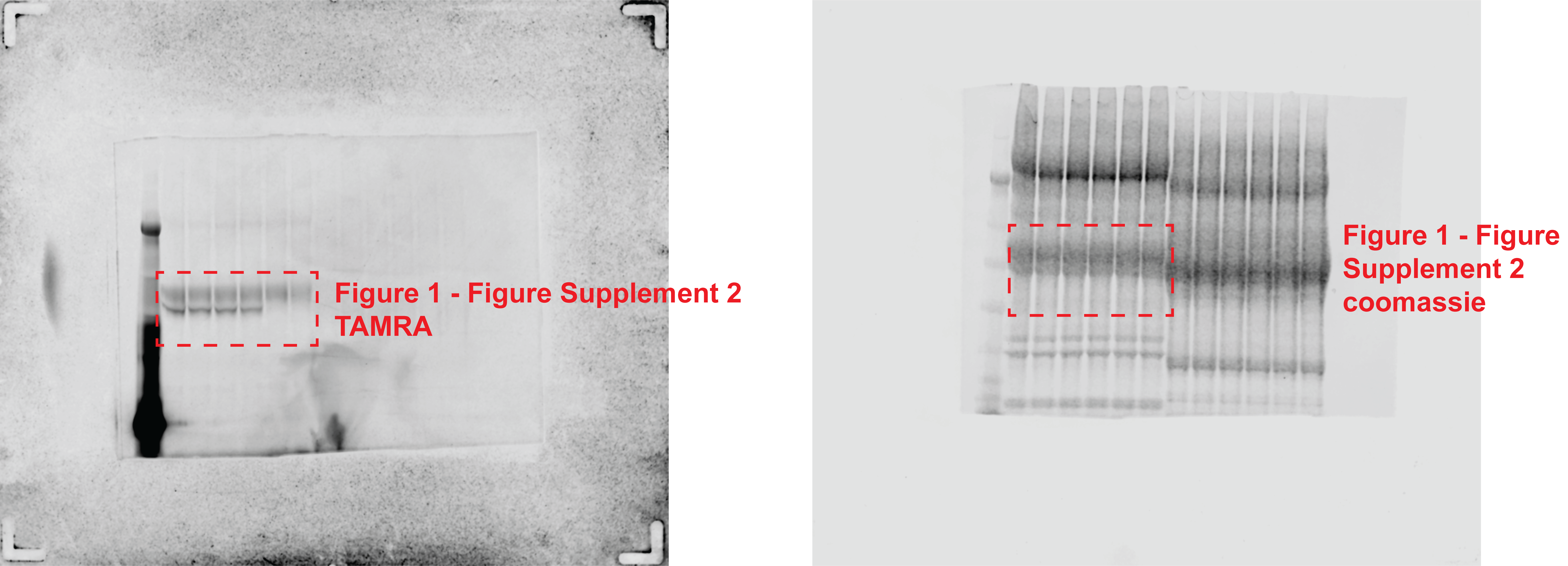

Supplement: Figure 1—figure supplement 2—source data 1. [file elife-85096-fig1-figsupp2-data1.zip › Figure1-fs2-sd1/Figure1_fs2_gelCroppingBounds.png]

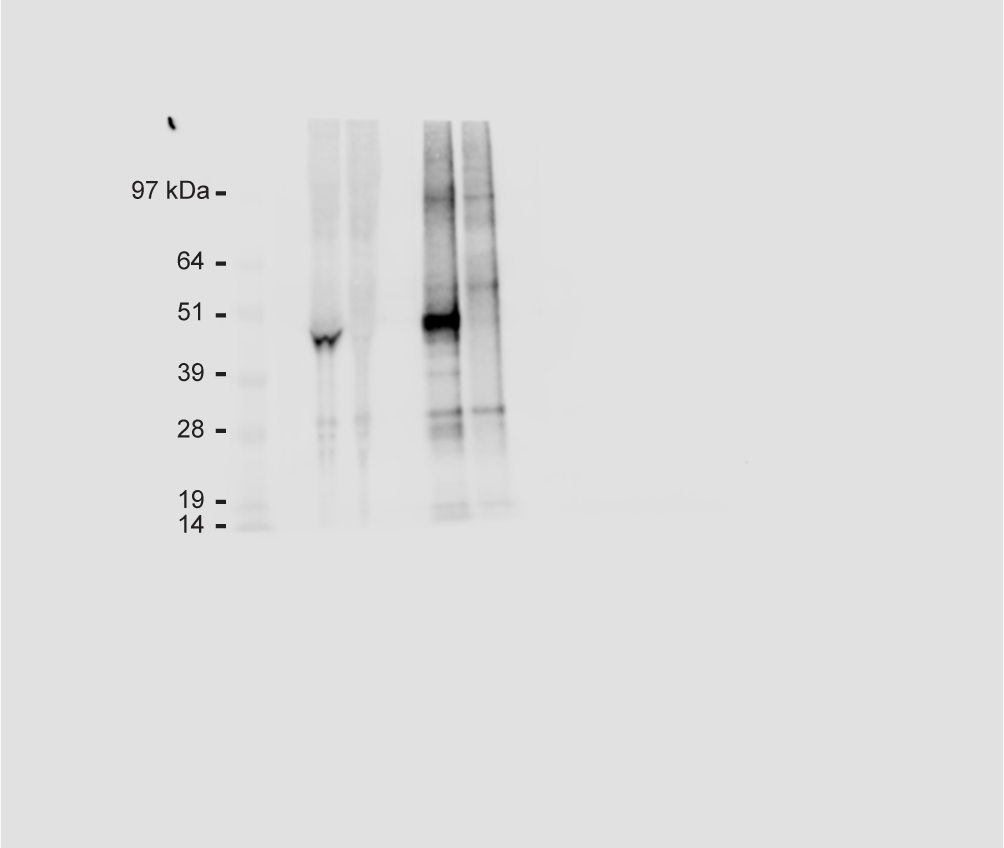

Supplement: Figure 2—source data 1. [file elife-85096-fig2-data1.zip › Figure2_sourcedata/2A_last3wells_strepBlot.tif]

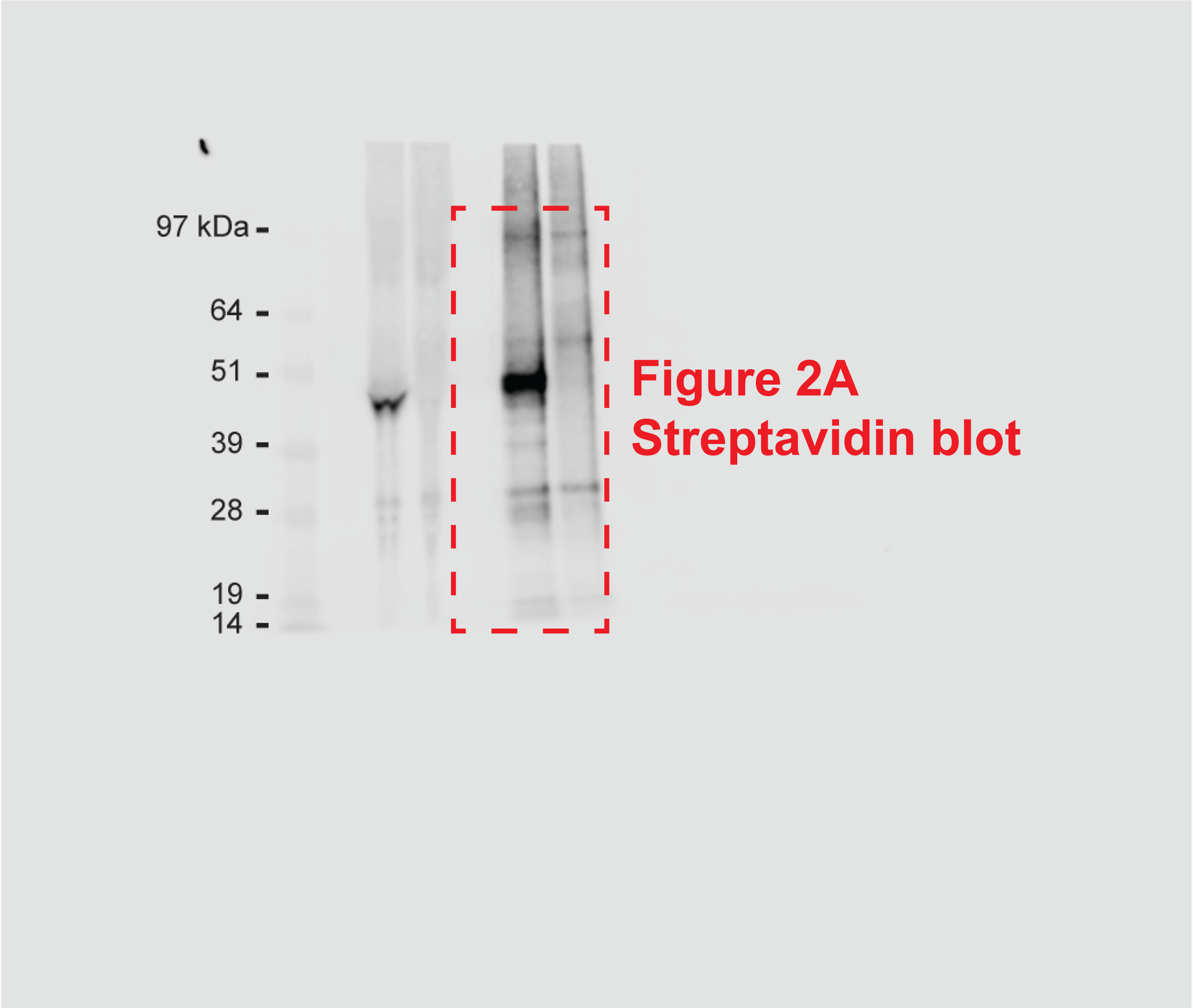

Supplement: Figure 2—source data 1. [file elife-85096-fig2-data1.zip › Figure2_sourcedata/Figure2_blotCroppingBounds.png]

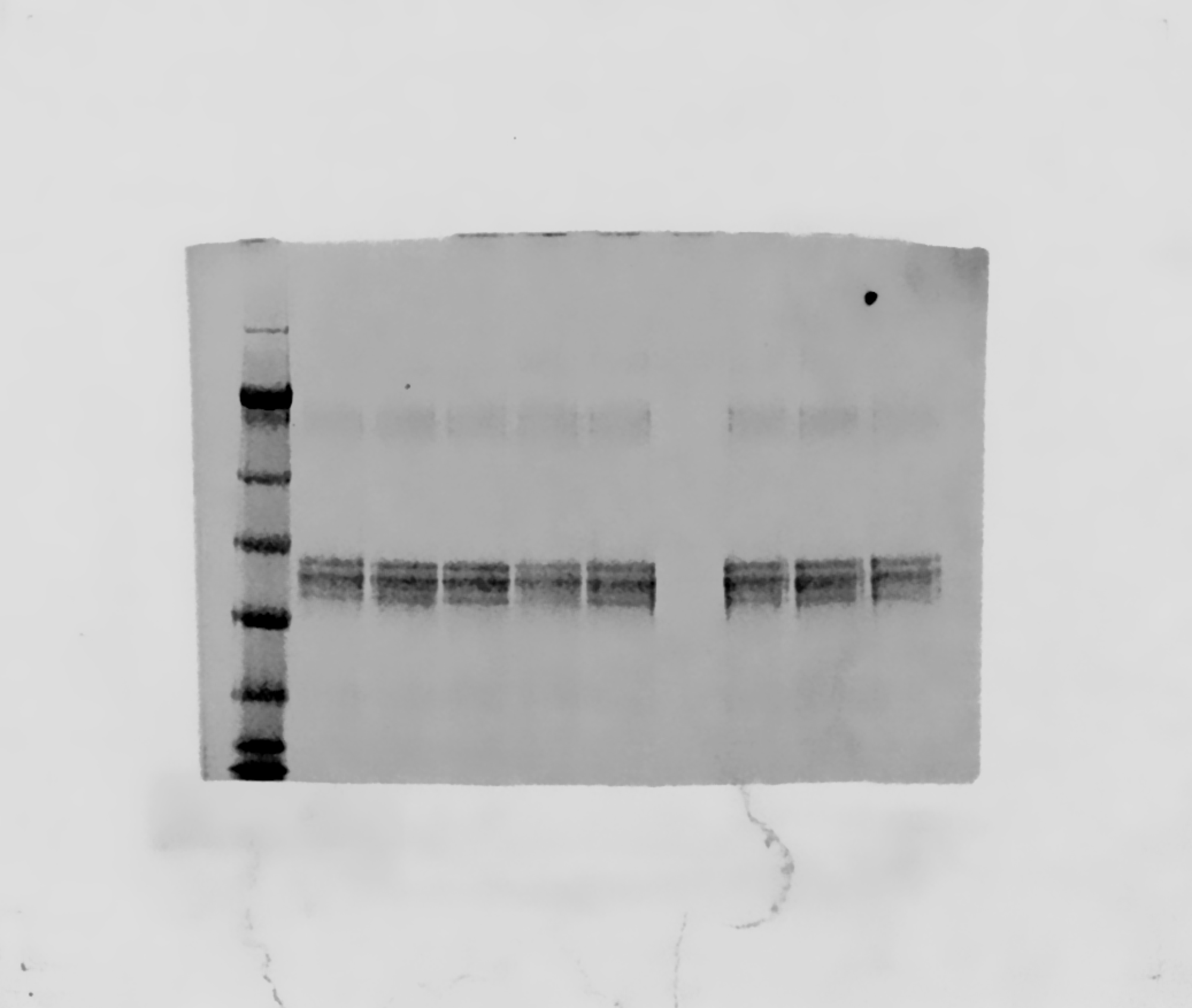

Supplement: Figure 4—source data 1. [file elife-85096-fig4-data1.zip › Figure4_sourcedata/4A_first5wells_coomassie.tif]

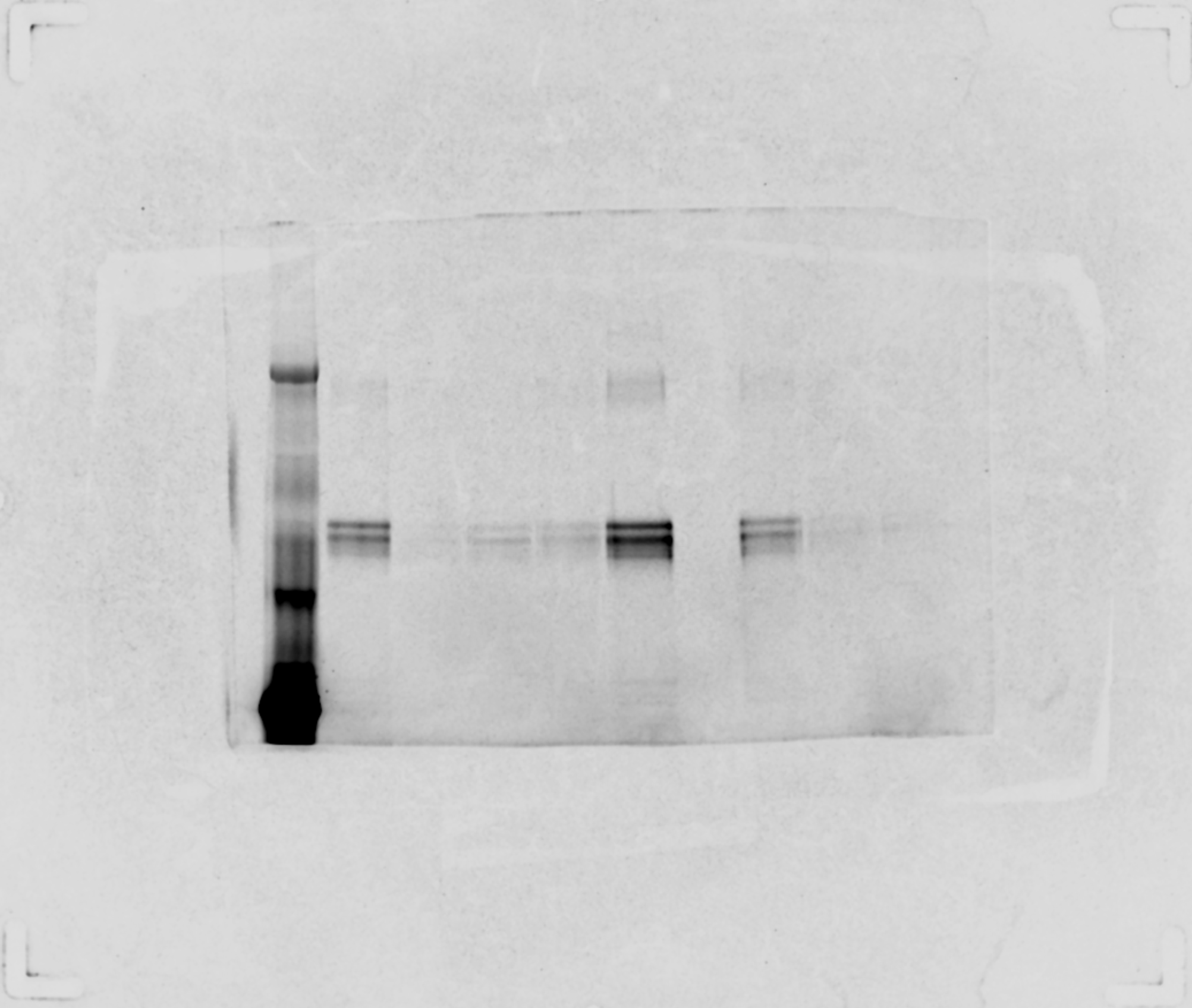

Supplement: Figure 4—source data 1. [file elife-85096-fig4-data1.zip › Figure4_sourcedata/4A_first5wells_TAMRA.tif]

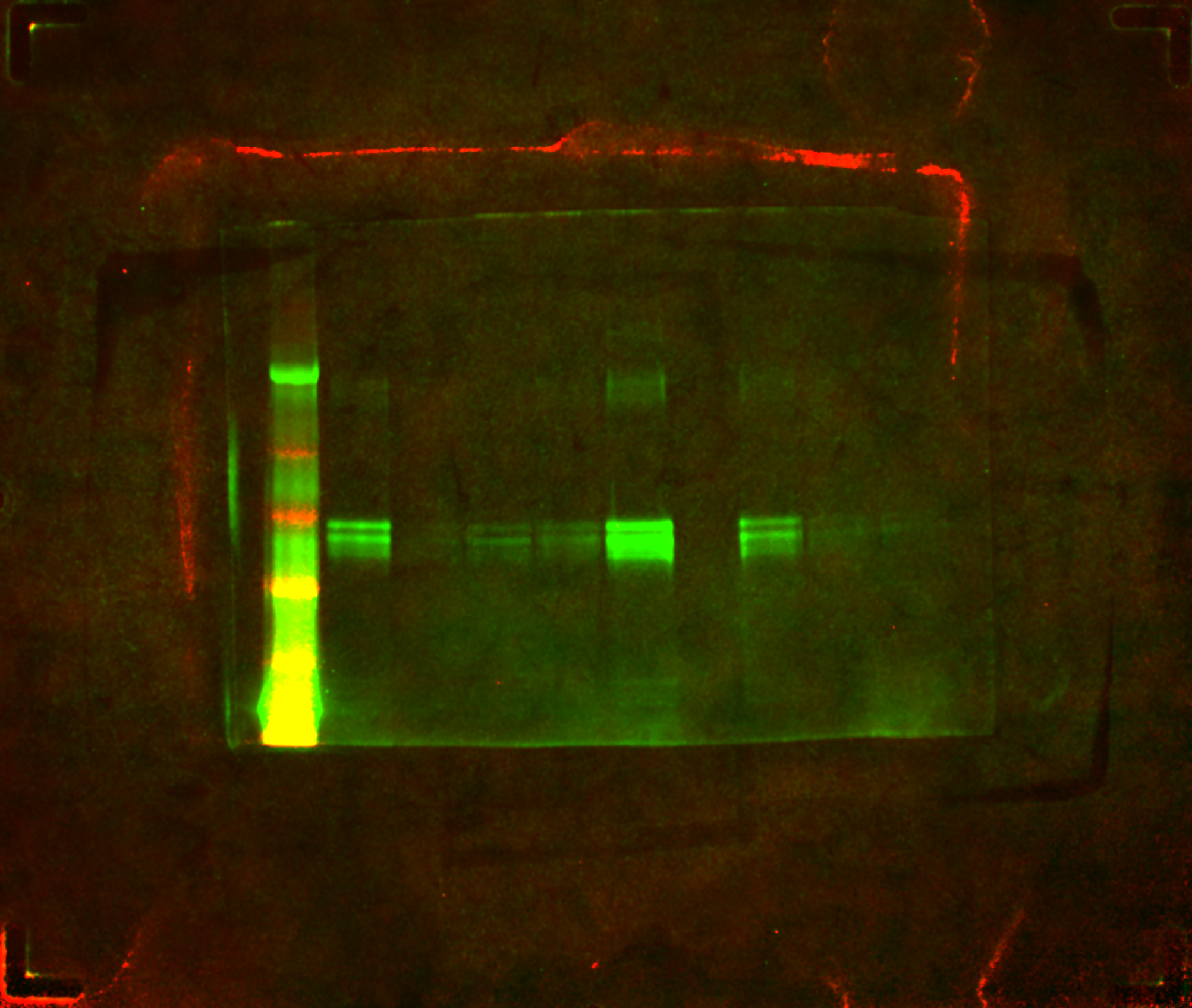

Supplement: Figure 4—source data 1. [file elife-85096-fig4-data1.zip › Figure4_sourcedata/4A_first5wells_TAMRAladder.tif]

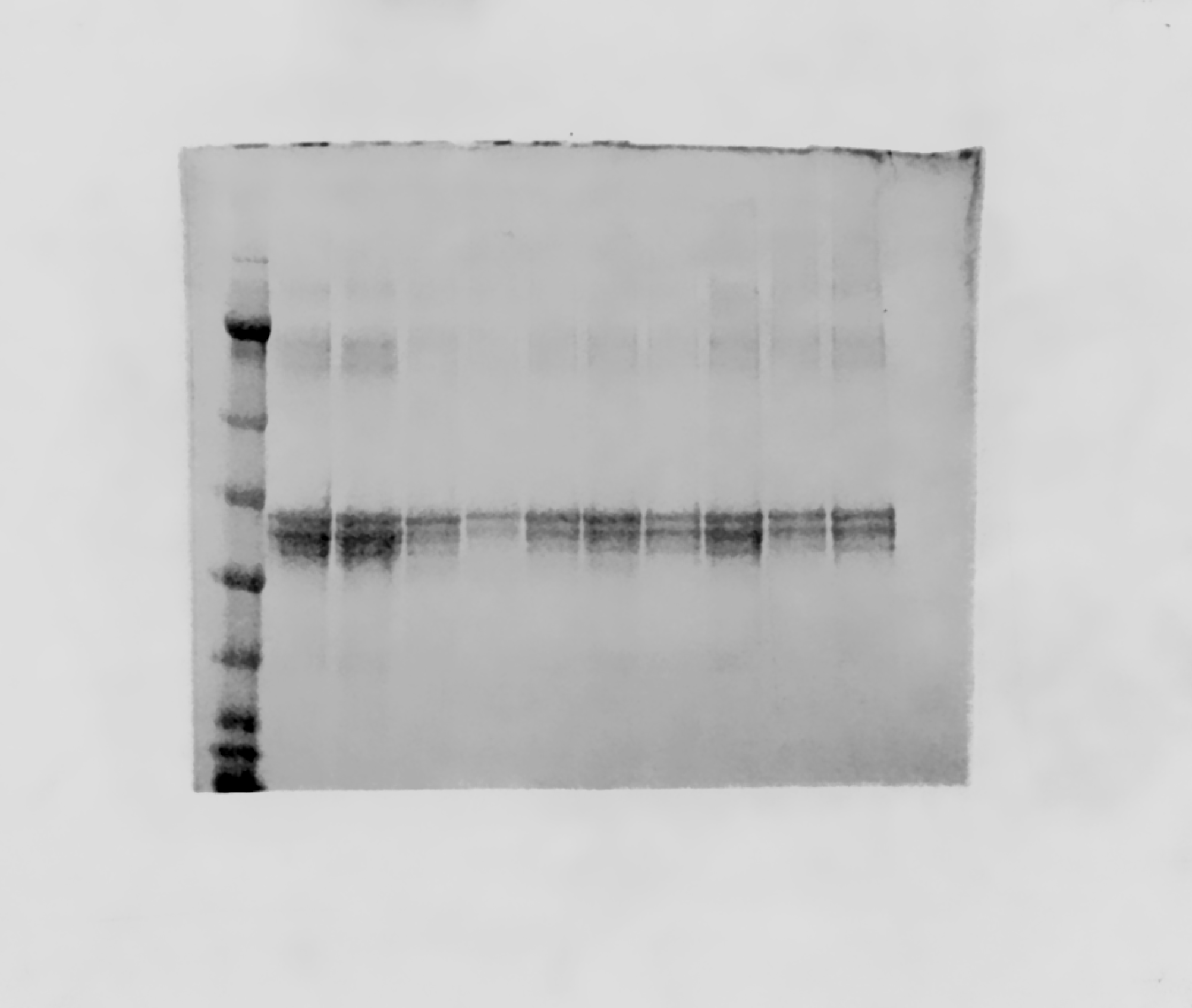

Supplement: Figure 4—source data 1. [file elife-85096-fig4-data1.zip › Figure4_sourcedata/4F_coomassie.tif]

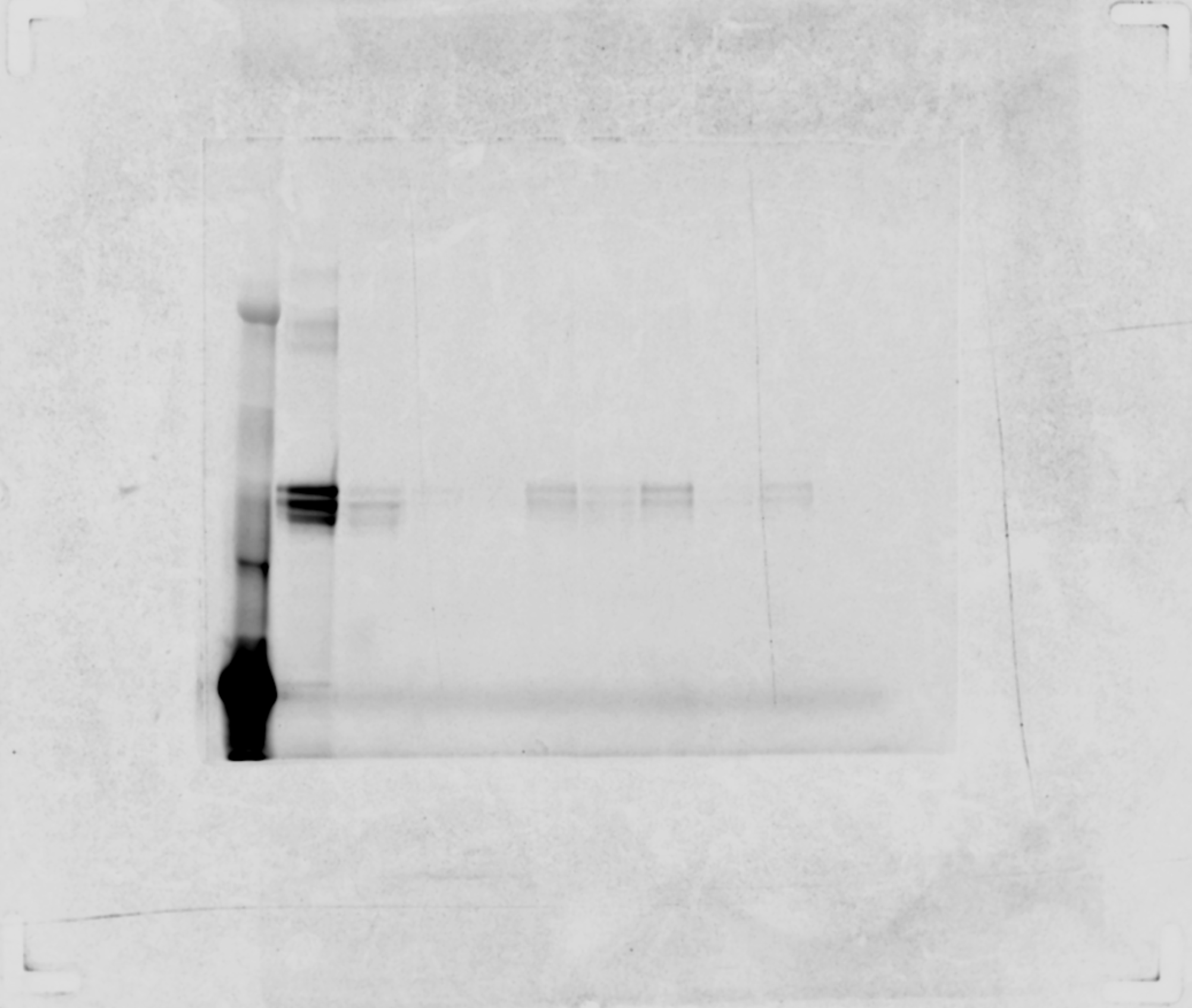

Supplement: Figure 4—source data 1. [file elife-85096-fig4-data1.zip › Figure4_sourcedata/4F_TAMRA.tif]

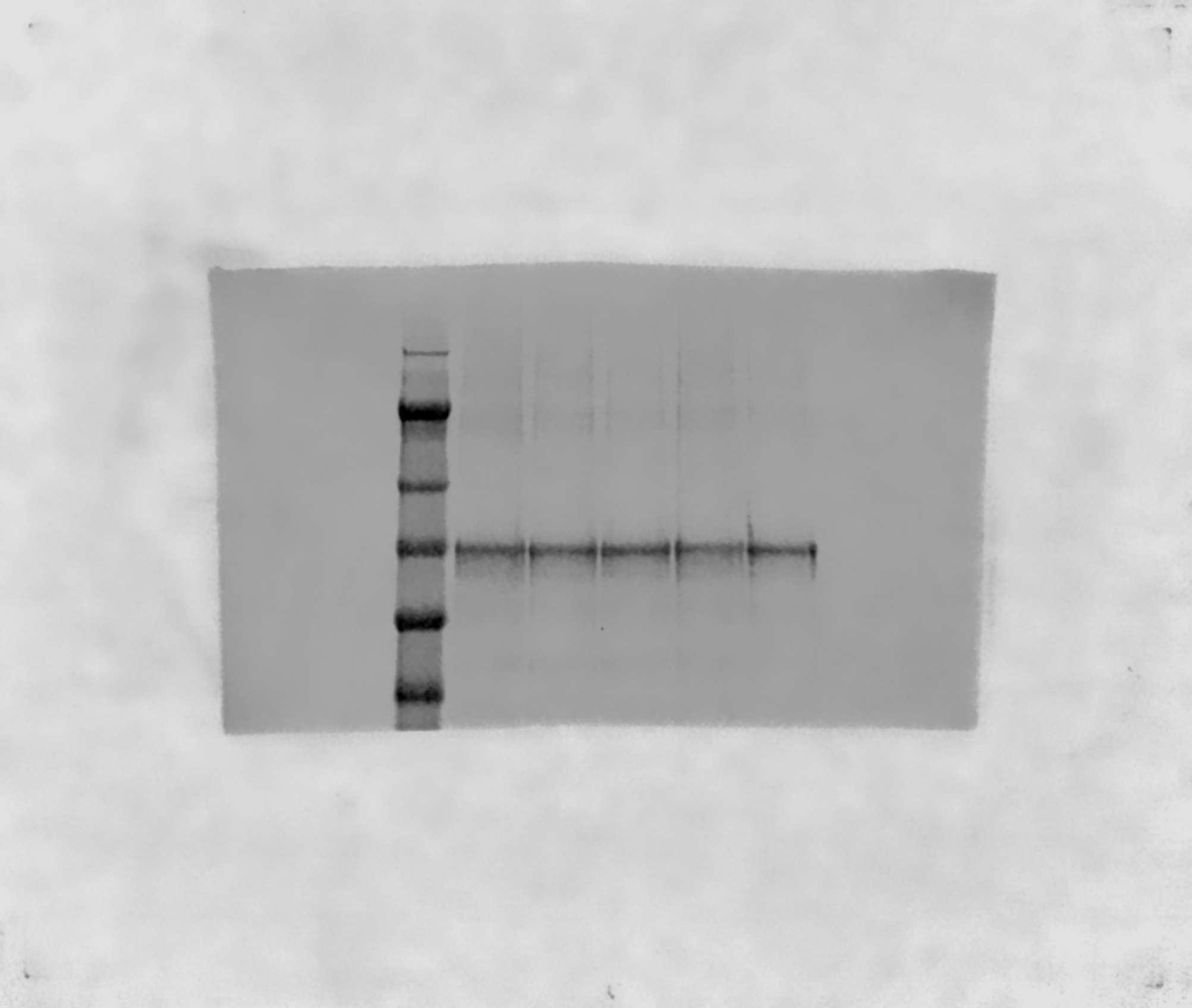

Supplement: Figure 4—source data 1. [file elife-85096-fig4-data1.zip › Figure4_sourcedata/4B_coomassie.tif]

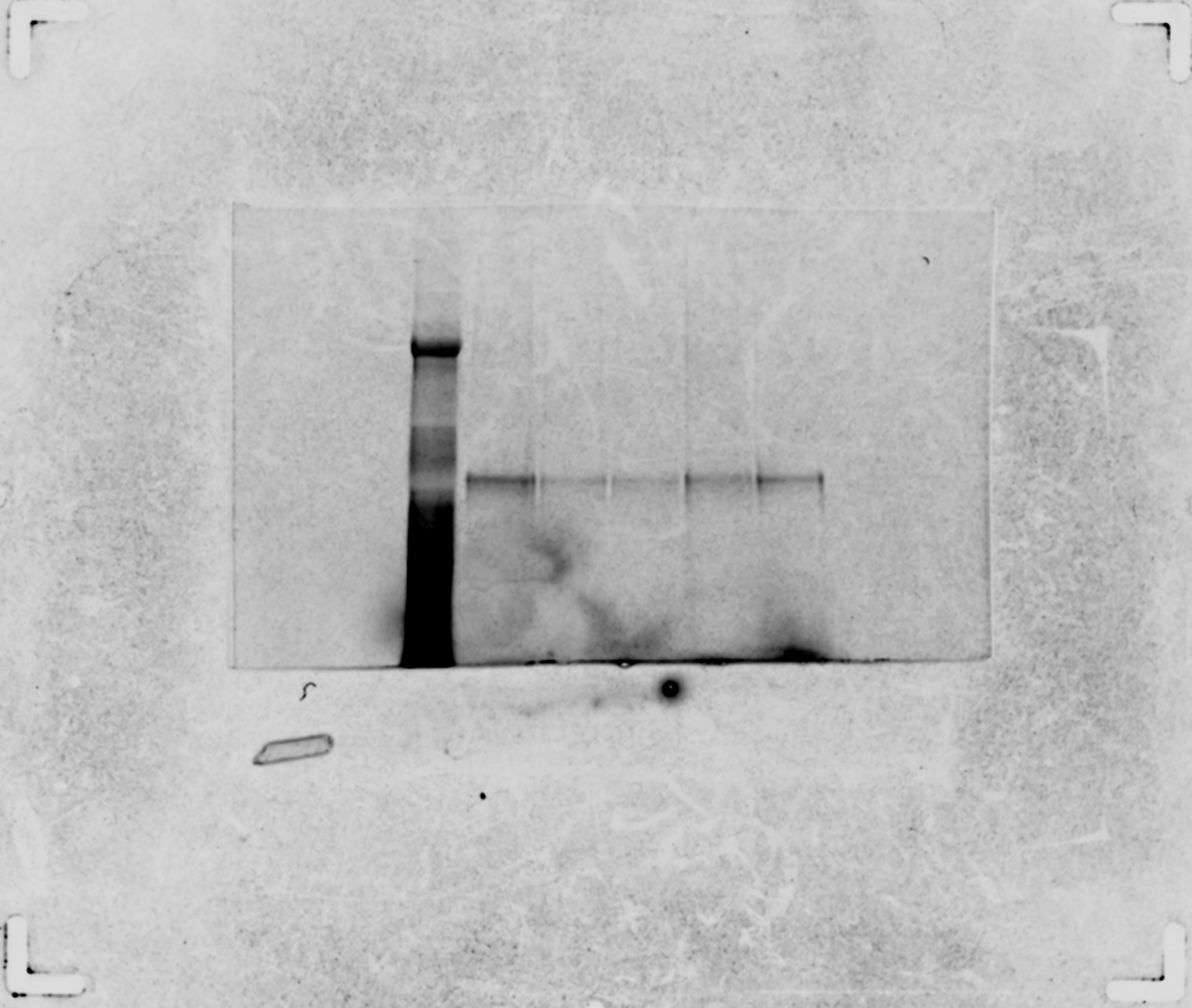

Supplement: Figure 4—source data 1. [file elife-85096-fig4-data1.zip › Figure4_sourcedata/4B_TAMRA.tif]

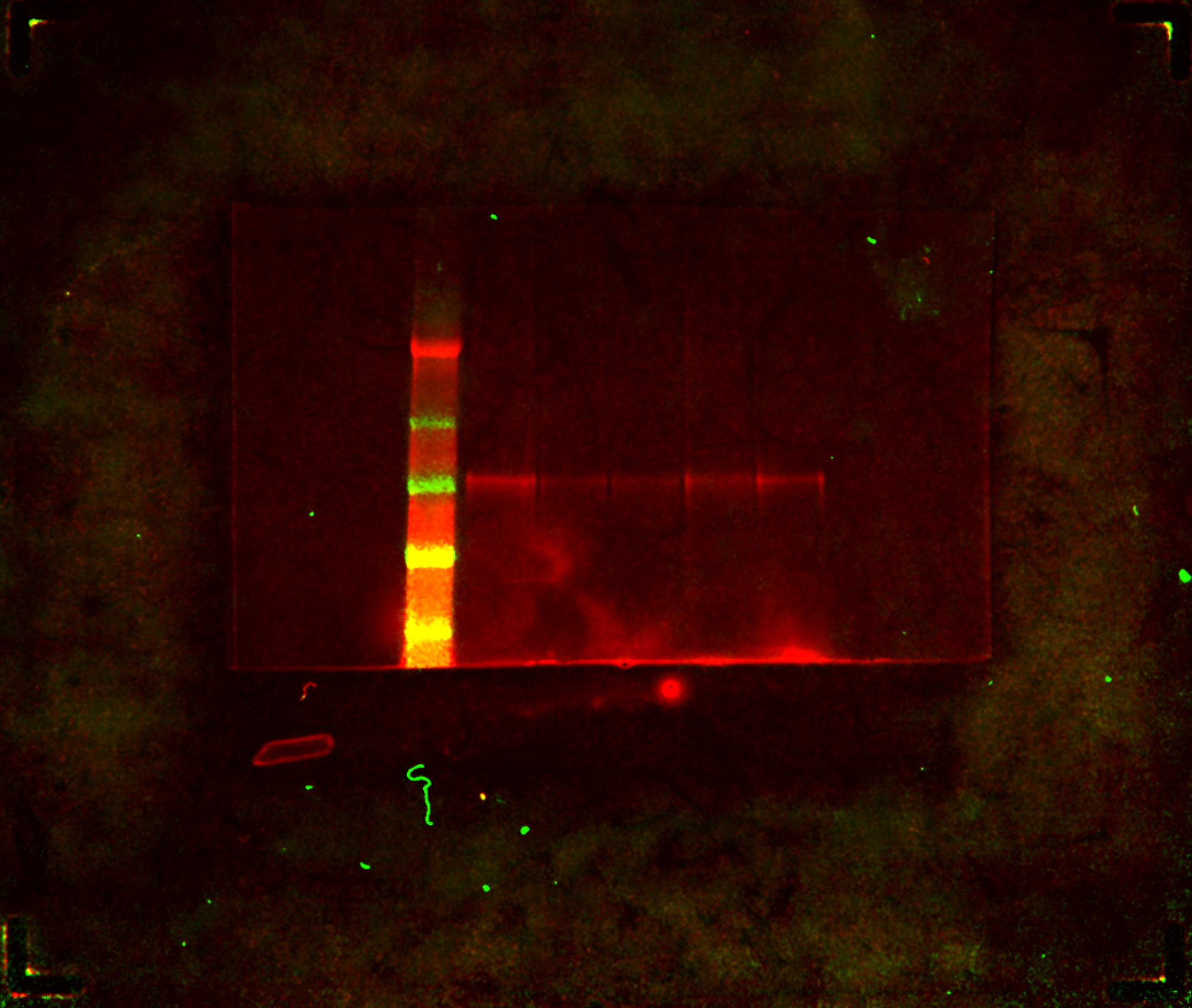

Supplement: Figure 4—source data 1. [file elife-85096-fig4-data1.zip › Figure4_sourcedata/4B_TAMRAladder.tif]

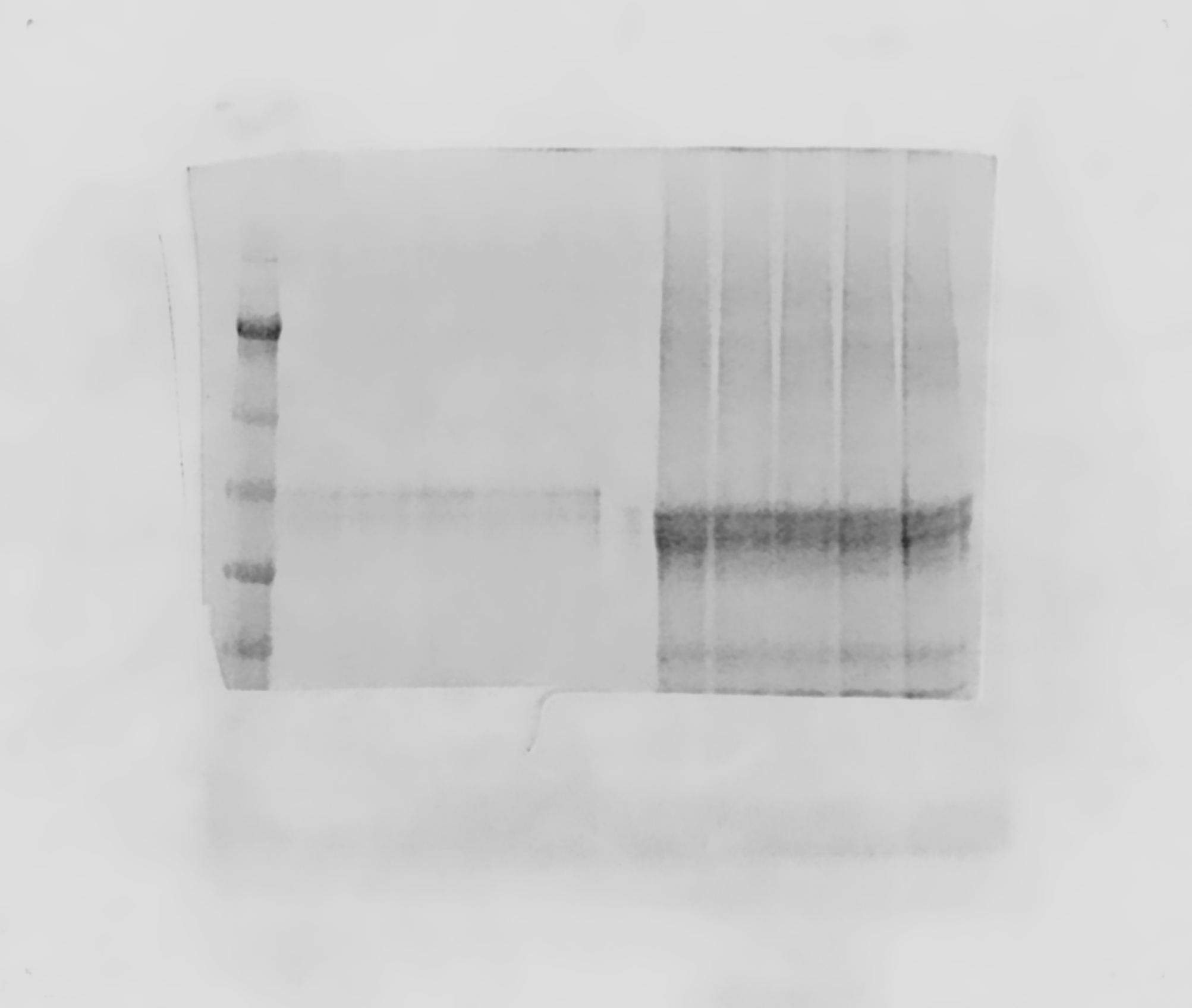

Supplement: Figure 4—source data 1. [file elife-85096-fig4-data1.zip › Figure4_sourcedata/4C_last5wells_coomassie.tif]

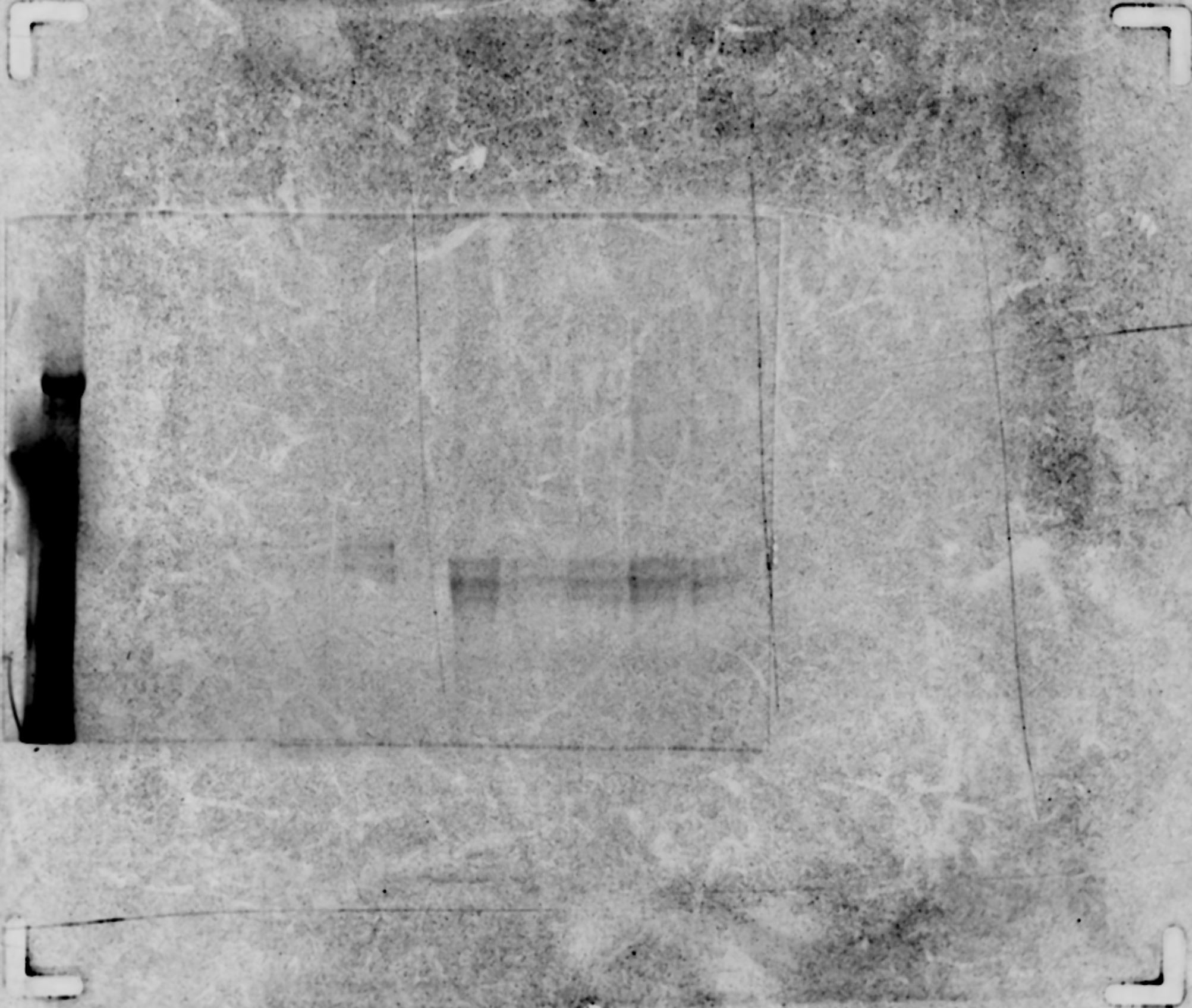

Supplement: Figure 4—source data 1. [file elife-85096-fig4-data1.zip › Figure4_sourcedata/4C_last5wells_TAMRA.tif]

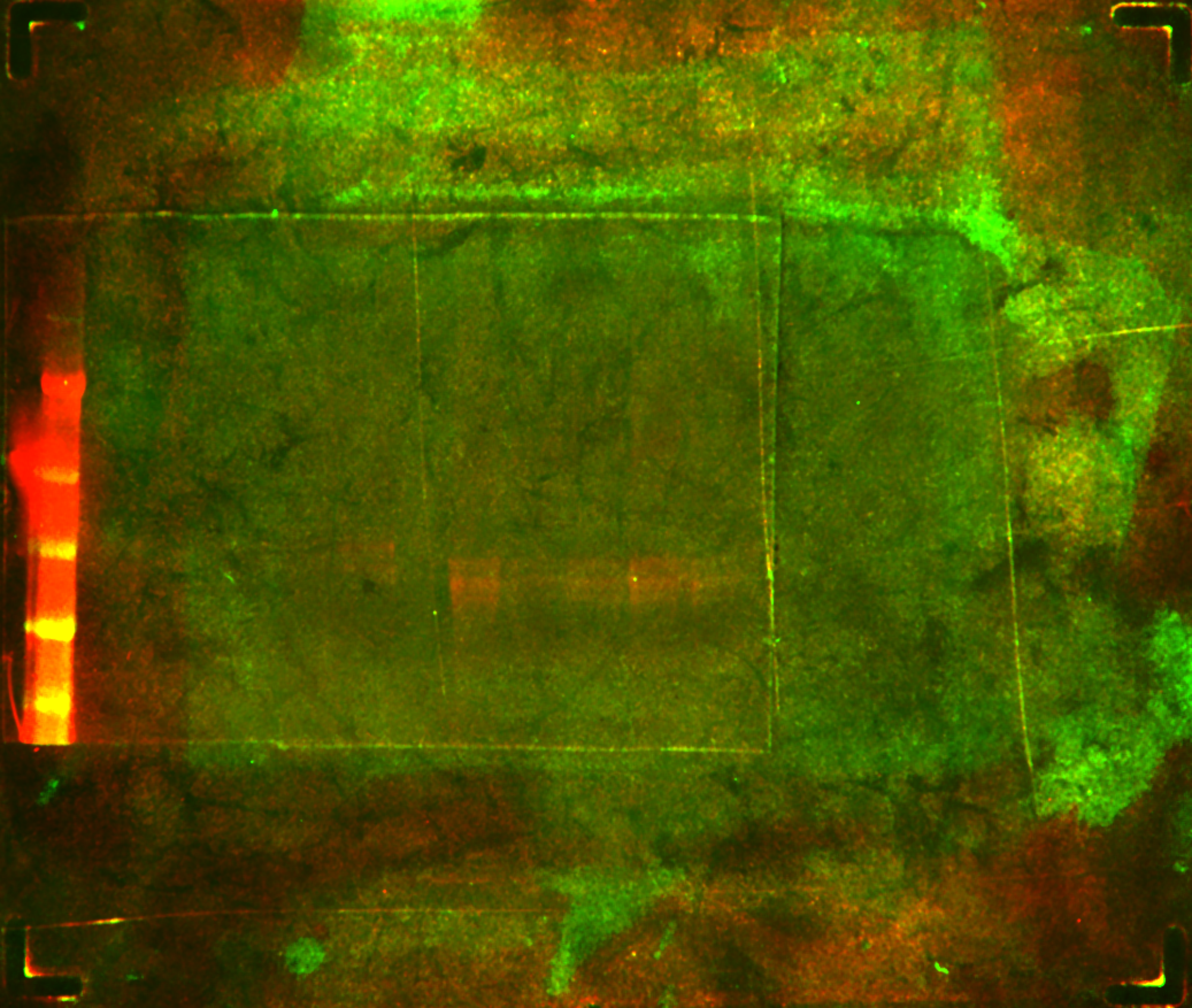

Supplement: Figure 4—source data 1. [file elife-85096-fig4-data1.zip › Figure4_sourcedata/4C_last5wells_TAMRAladder.tif]

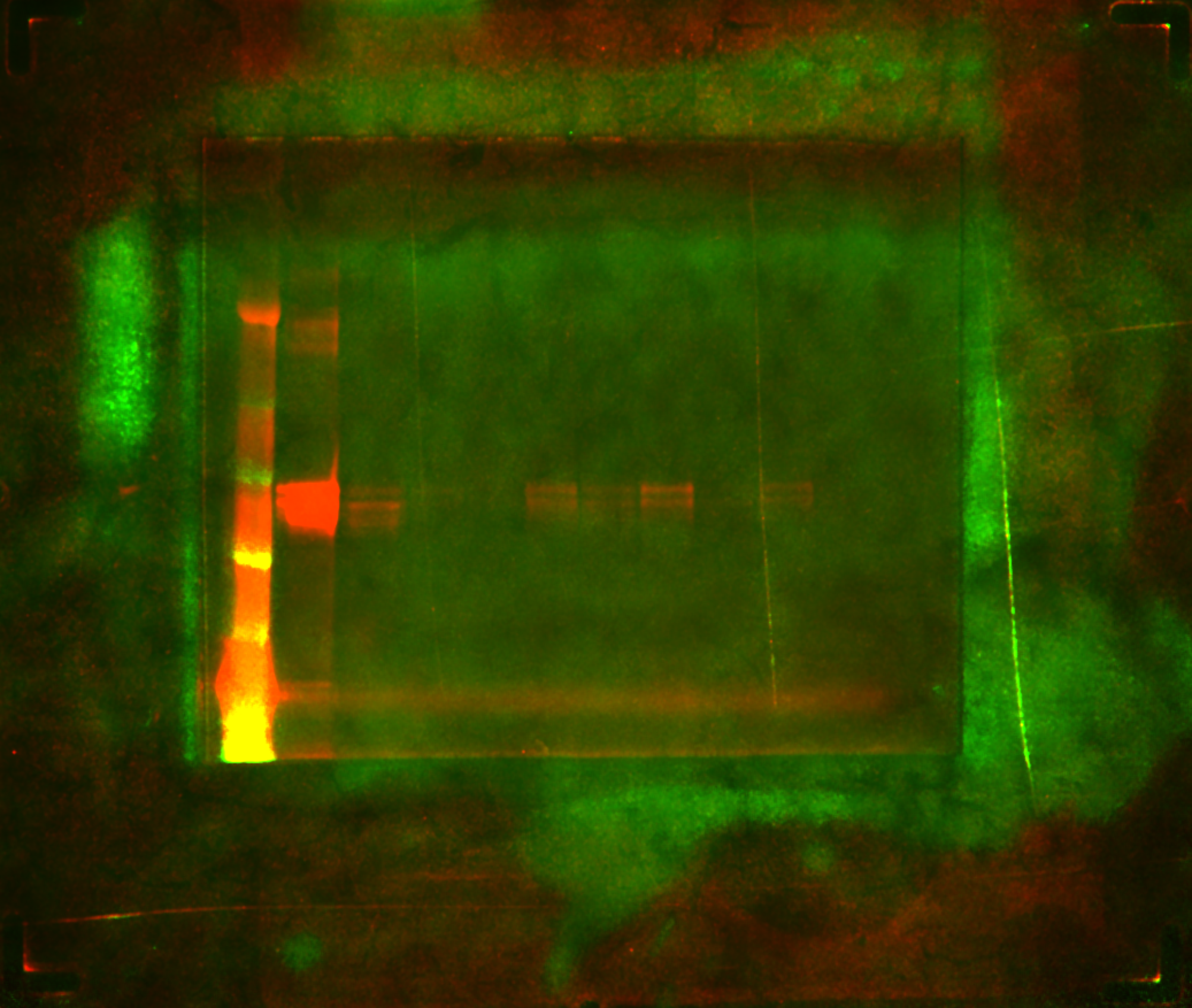

Supplement: Figure 4—source data 1. [file elife-85096-fig4-data1.zip › Figure4_sourcedata/4F_TAMRAladder.tif]

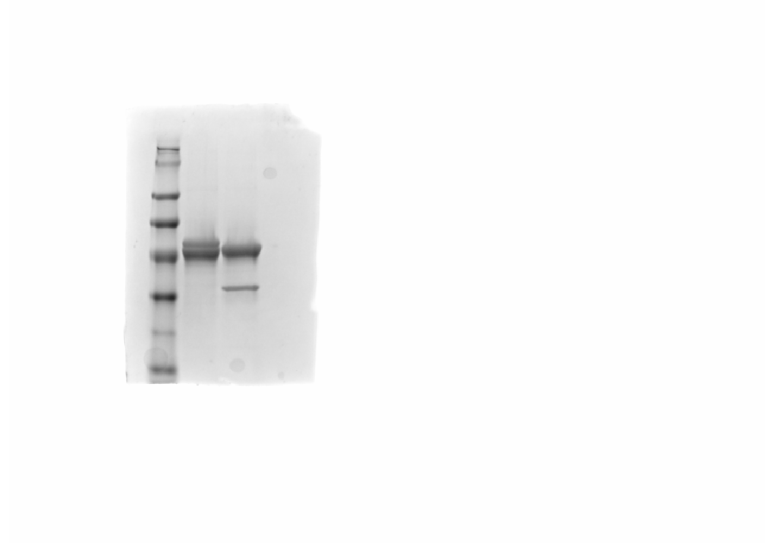

Supplement: Figure 4—figure supplement 1—source data 1. [file elife-85096-fig4-figsupp1-data1.zip › Figure4-fs1-sd1/S1C_coomassie.tif]

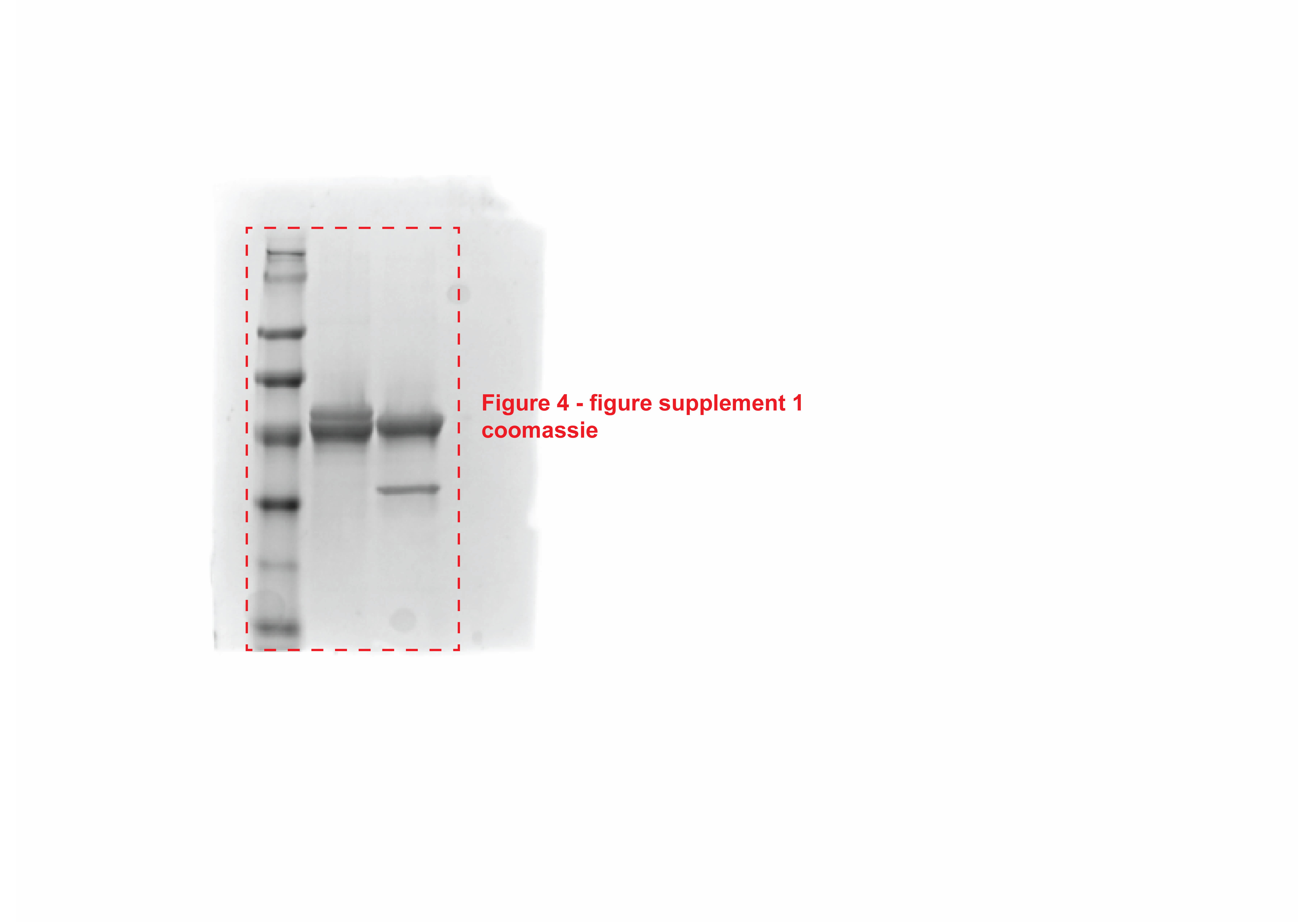

Supplement: Figure 4—figure supplement 1—source data 1. [file elife-85096-fig4-figsupp1-data1.zip › Figure4-fs1-sd1/Figure4_fs1_labeledGels.png]

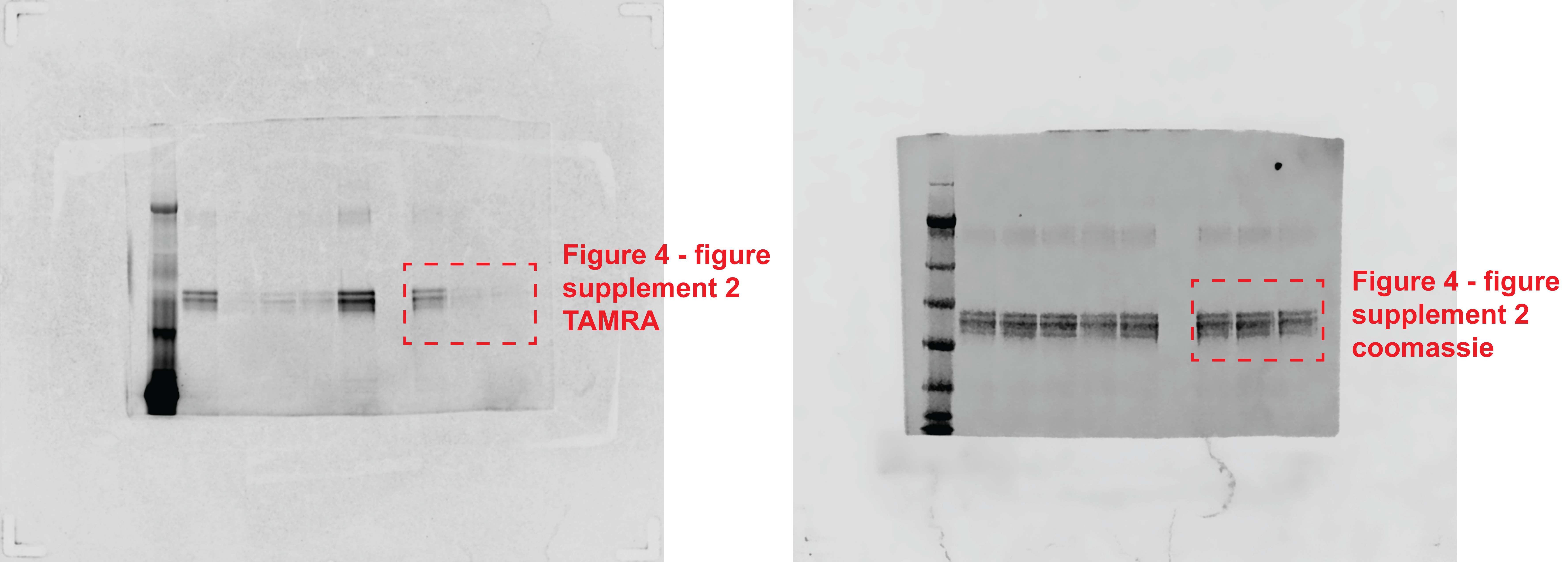

Supplement: Figure 4—figure supplement 2—source data 1. [file elife-85096-fig4-figsupp2-data1.zip › Figure4-fs2-sd1/Figure4_fs2_gelCroppingBounds.png]

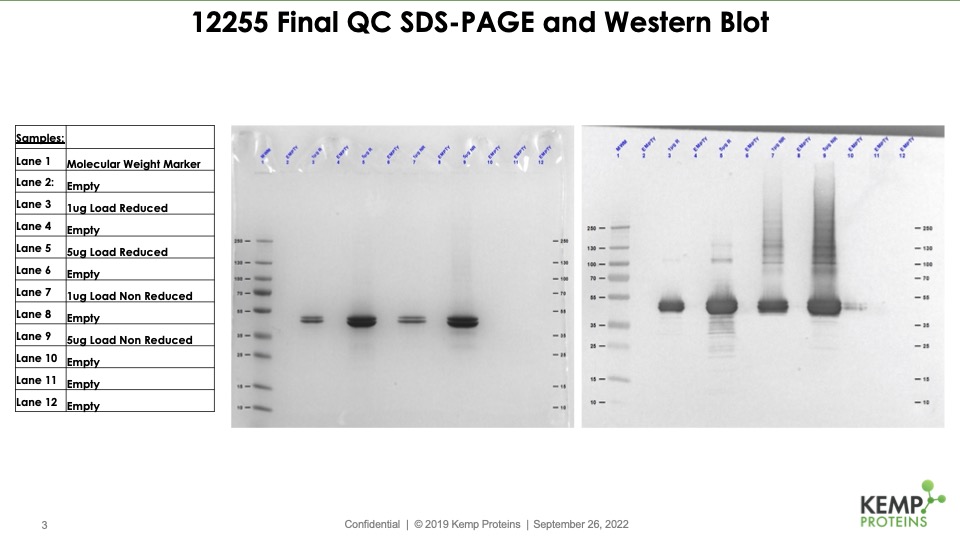

Supplement: Figure 4—figure supplement 3—source data 1. [file elife-85096-fig4-figsupp3-data1.zip › Figure4-fs3-sd1/S4A_rightblot_well3_well7__S4B_leftgel_well3_well7.jpg]

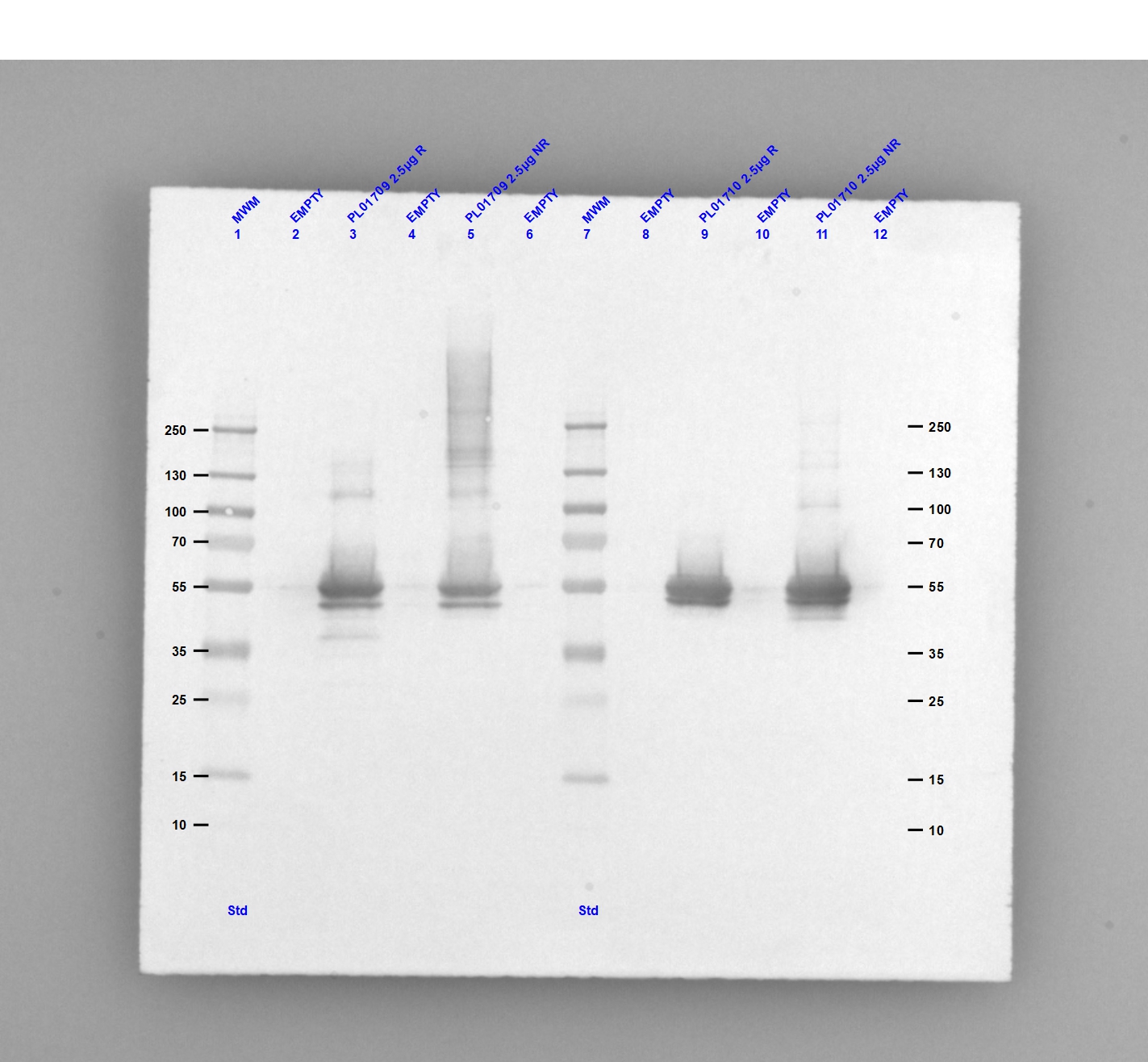

Supplement: Figure 4—figure supplement 3—source data 1. [file elife-85096-fig4-figsupp3-data1.zip › Figure4-fs3-sd1/S4C_well3_well5__S4E_well9_well11.jpg]

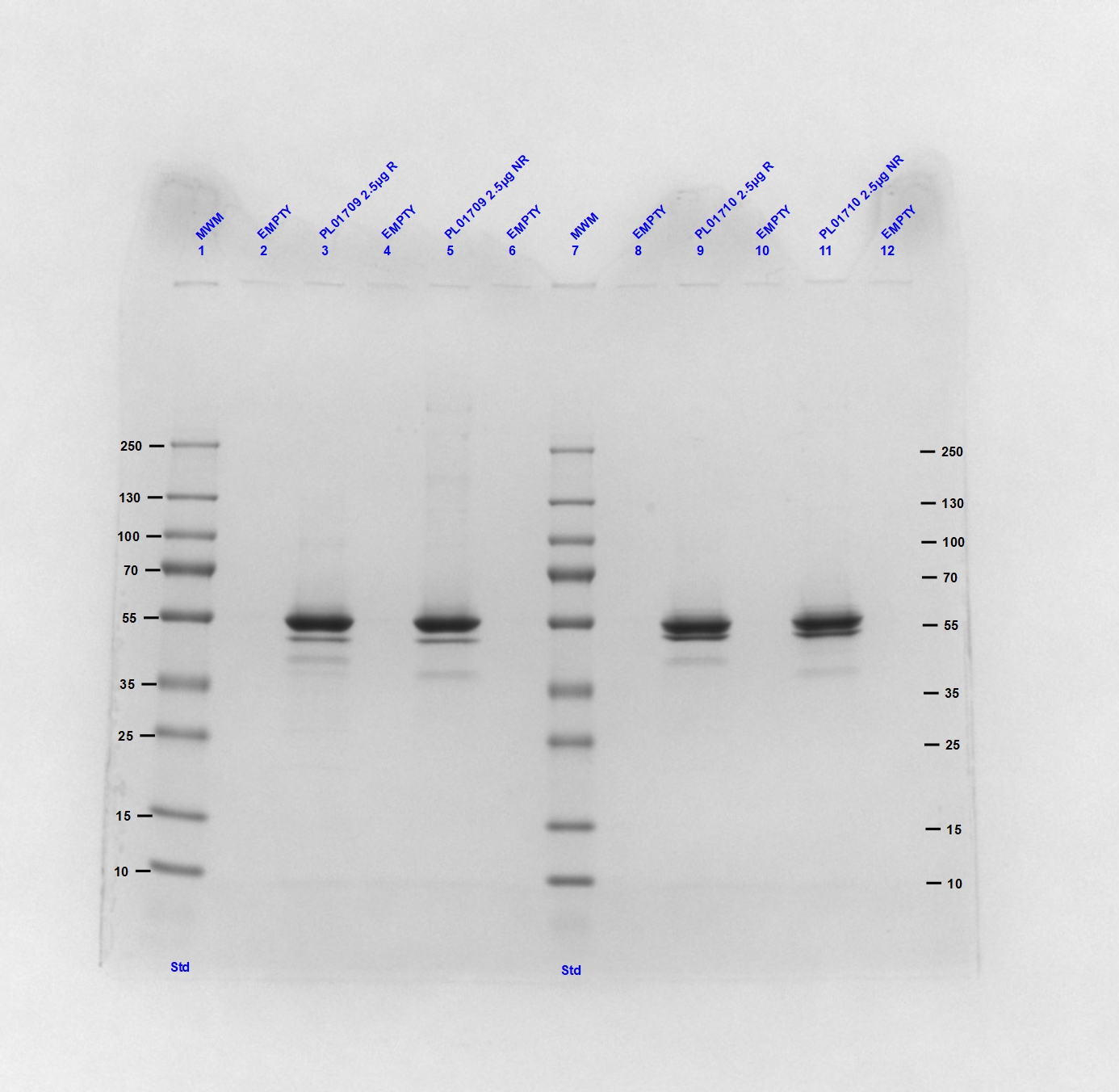

Supplement: Figure 4—figure supplement 3—source data 1. [file elife-85096-fig4-figsupp3-data1.zip › Figure4-fs3-sd1/S4D_well3_well5__S4F_well9_well11.jpg]

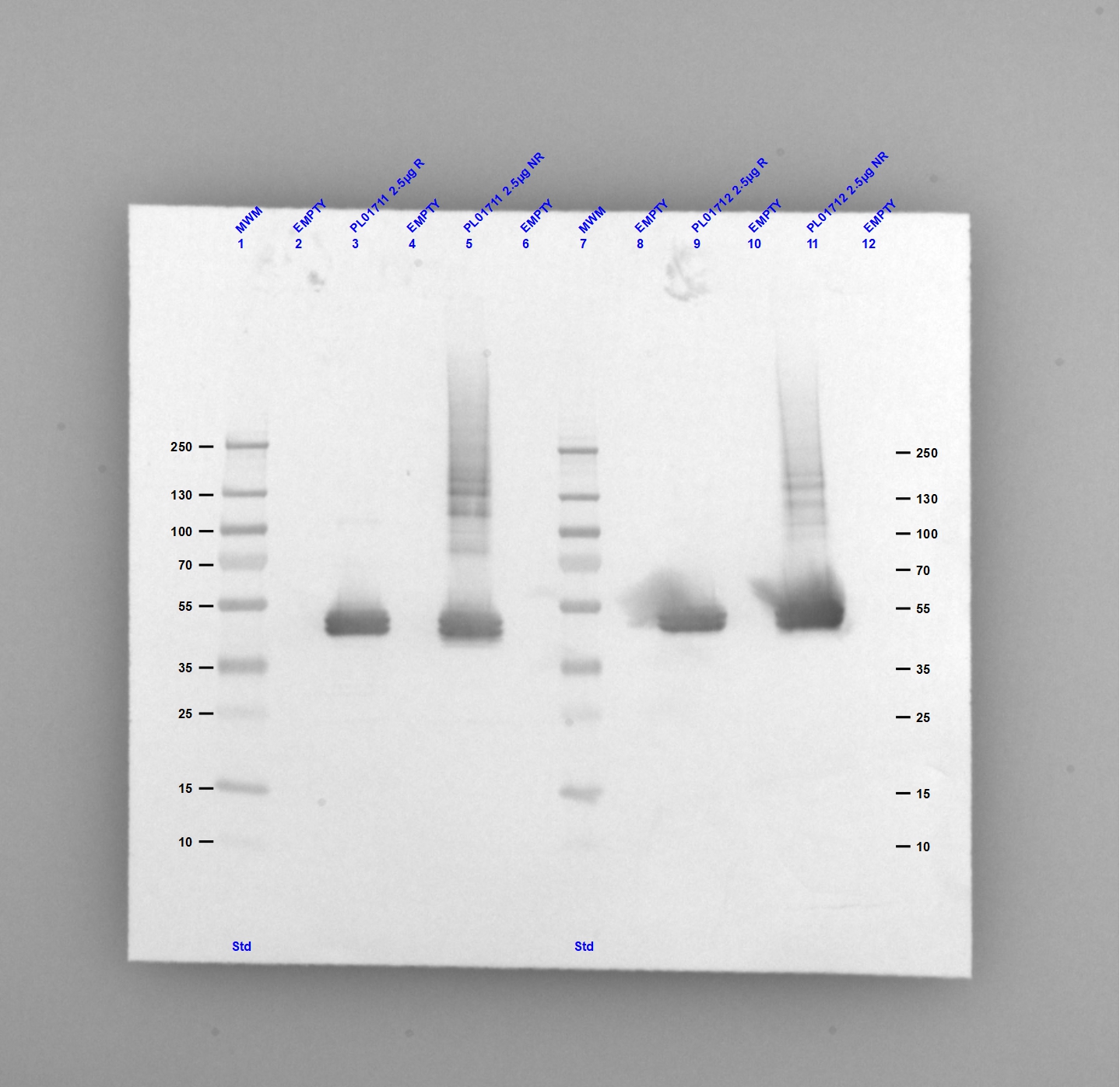

Supplement: Figure 4—figure supplement 3—source data 1. [file elife-85096-fig4-figsupp3-data1.zip › Figure4-fs3-sd1/S4G_well3_well5__S4I_well9_well11.jpg]

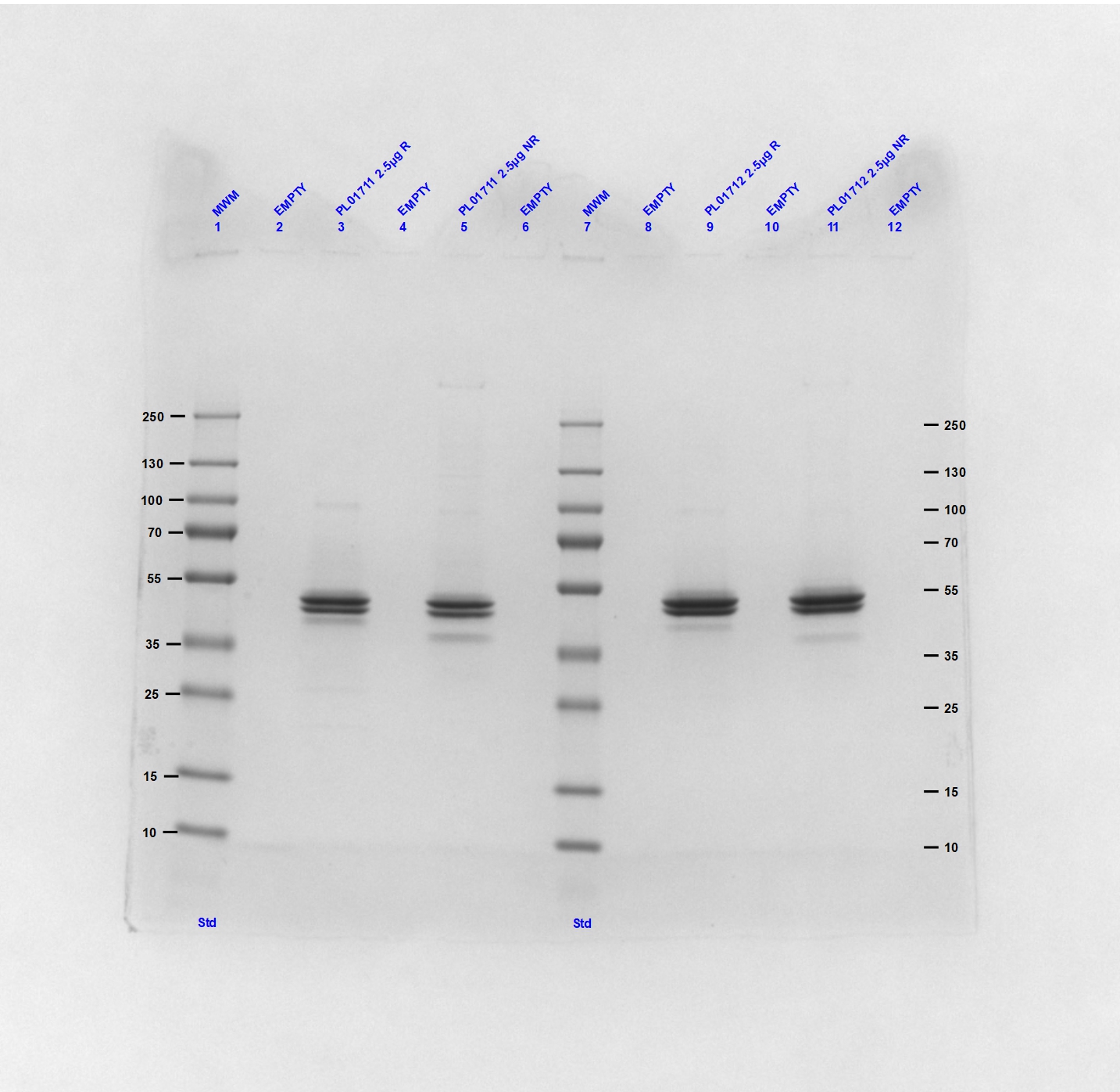

Supplement: Figure 4—figure supplement 3—source data 1. [file elife-85096-fig4-figsupp3-data1.zip › Figure4-fs3-sd1/S4H_well3_well5__S4J_well9_well11.jpg]

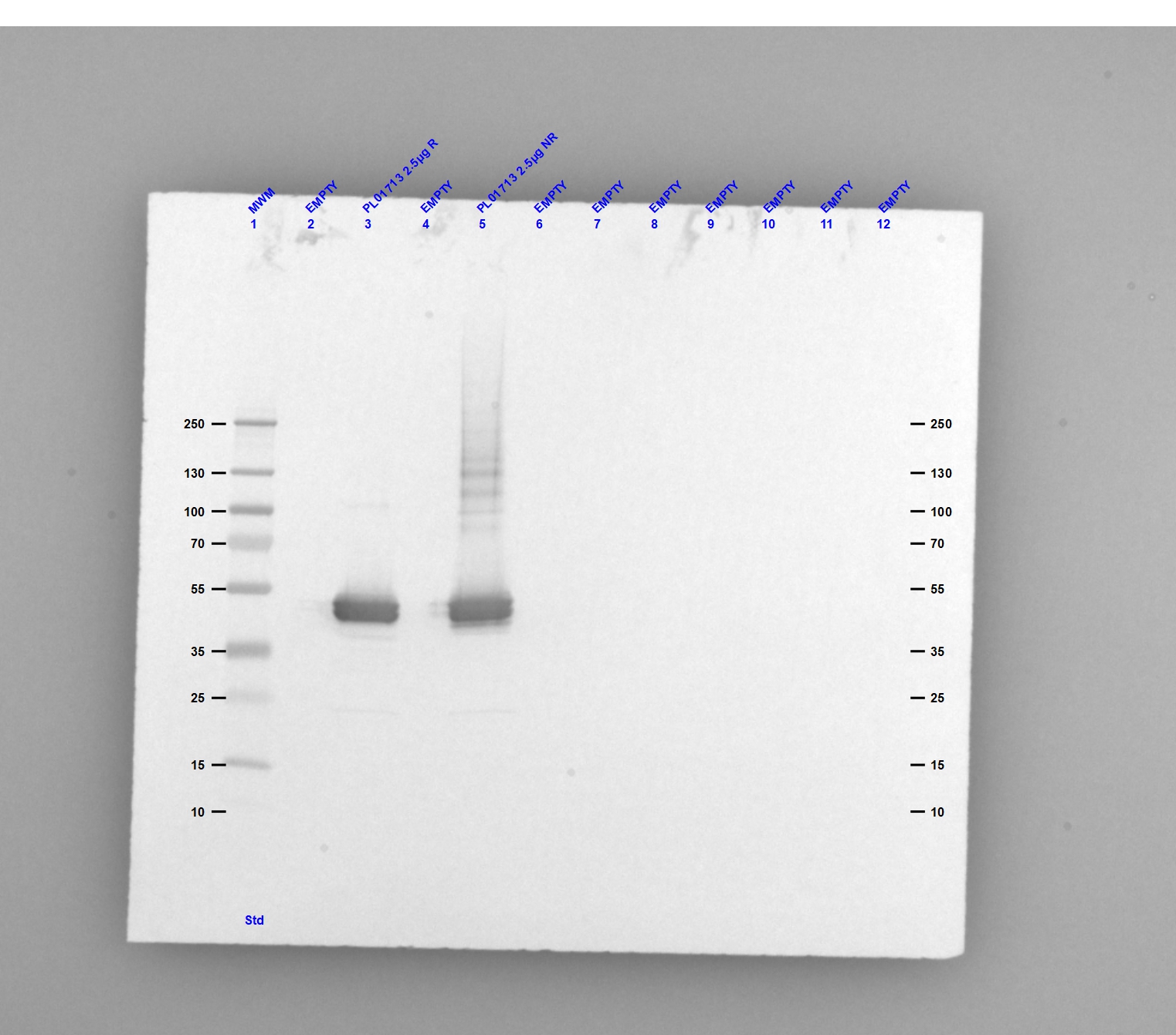

Supplement: Figure 4—figure supplement 3—source data 1. [file elife-85096-fig4-figsupp3-data1.zip › Figure4-fs3-sd1/S4K_well3_well5.jpg]

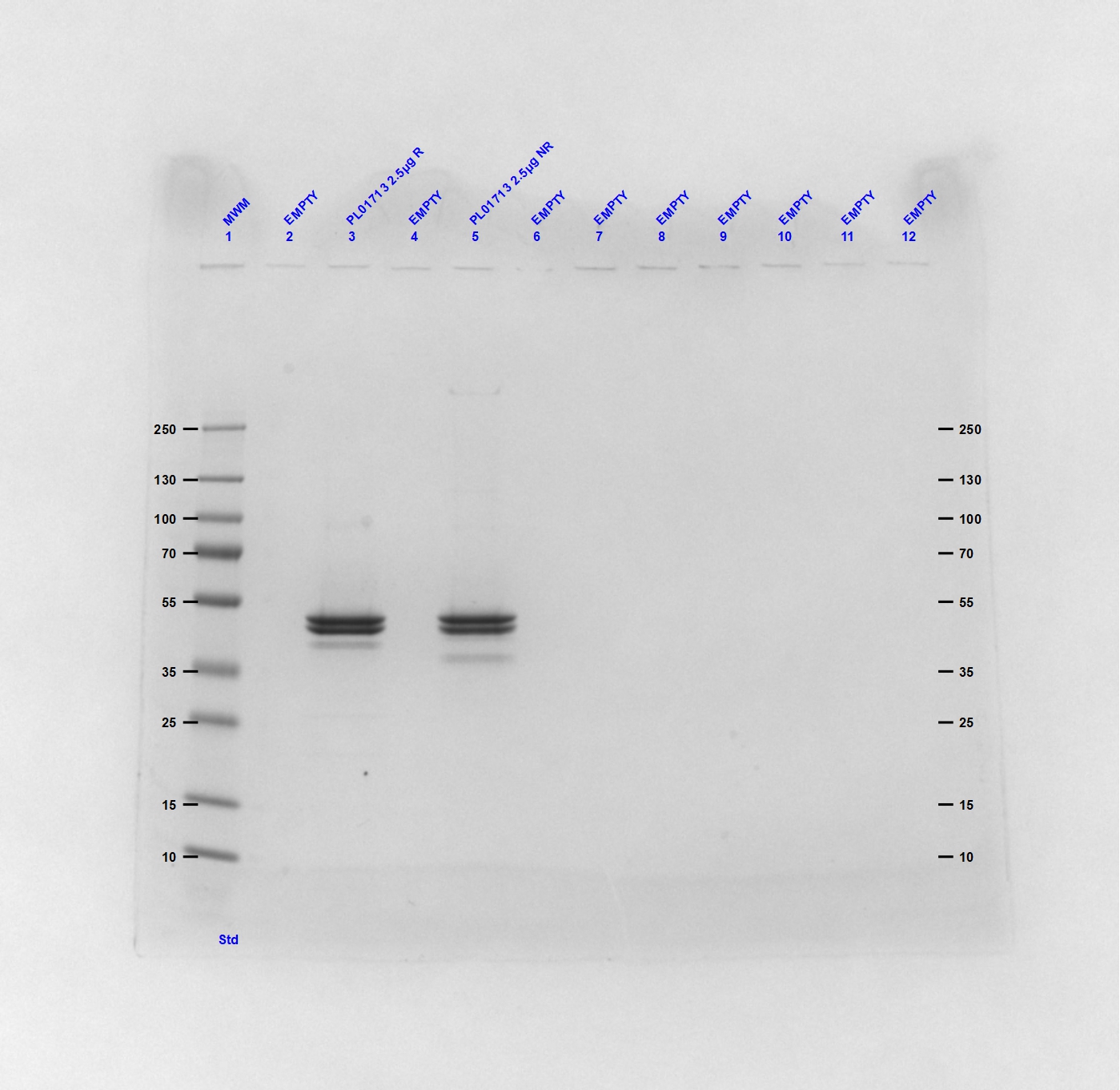

Supplement: Figure 4—figure supplement 3—source data 1. [file elife-85096-fig4-figsupp3-data1.zip › Figure4-fs3-sd1/S4L_well3_well5.jpg]

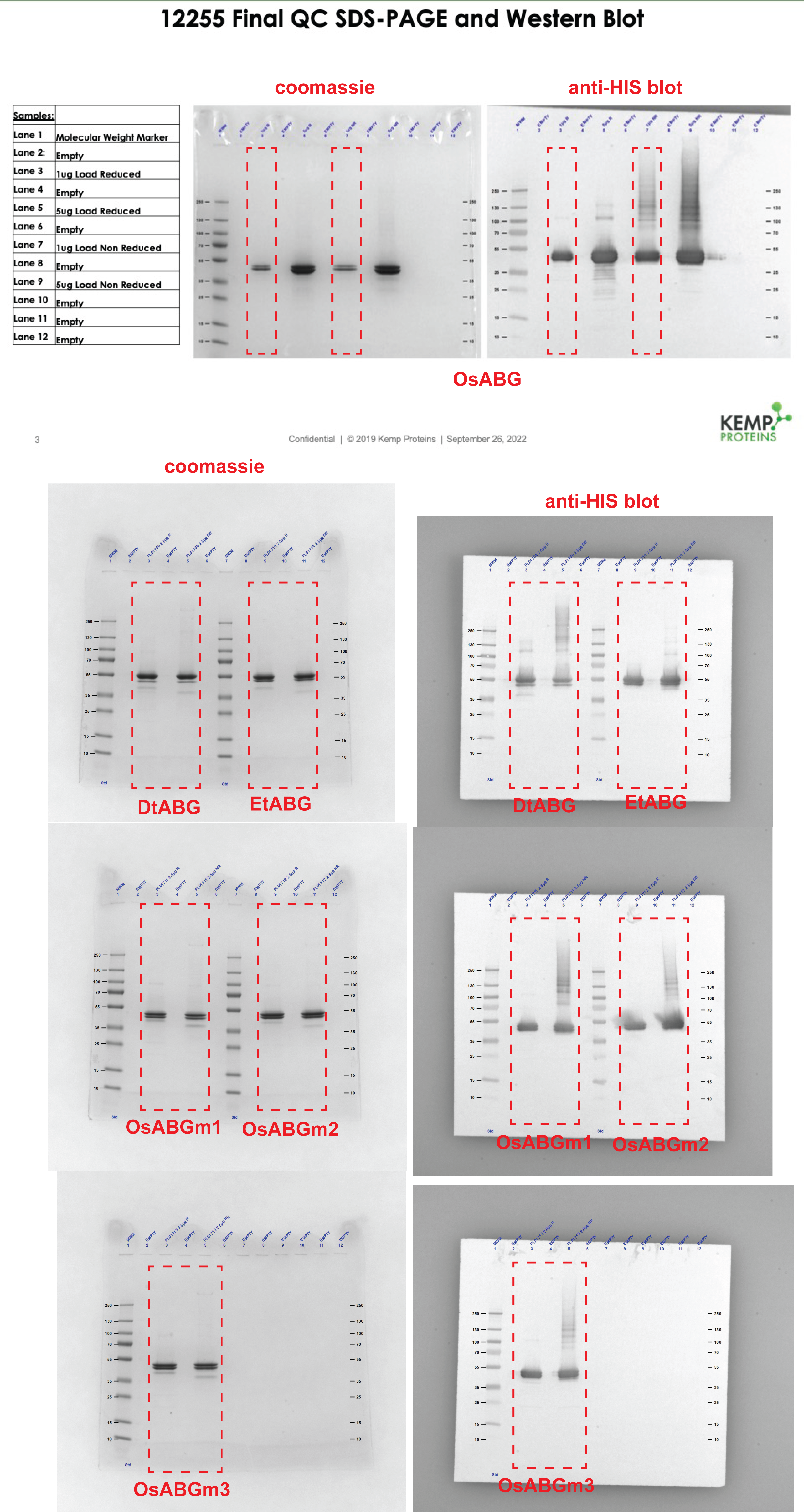

Supplement: Figure 4—figure supplement 3—source data 1. [file elife-85096-fig4-figsupp3-data1.zip › Figure4-fs3-sd1/Figure4_fs3_gelCroppingBounds.png]

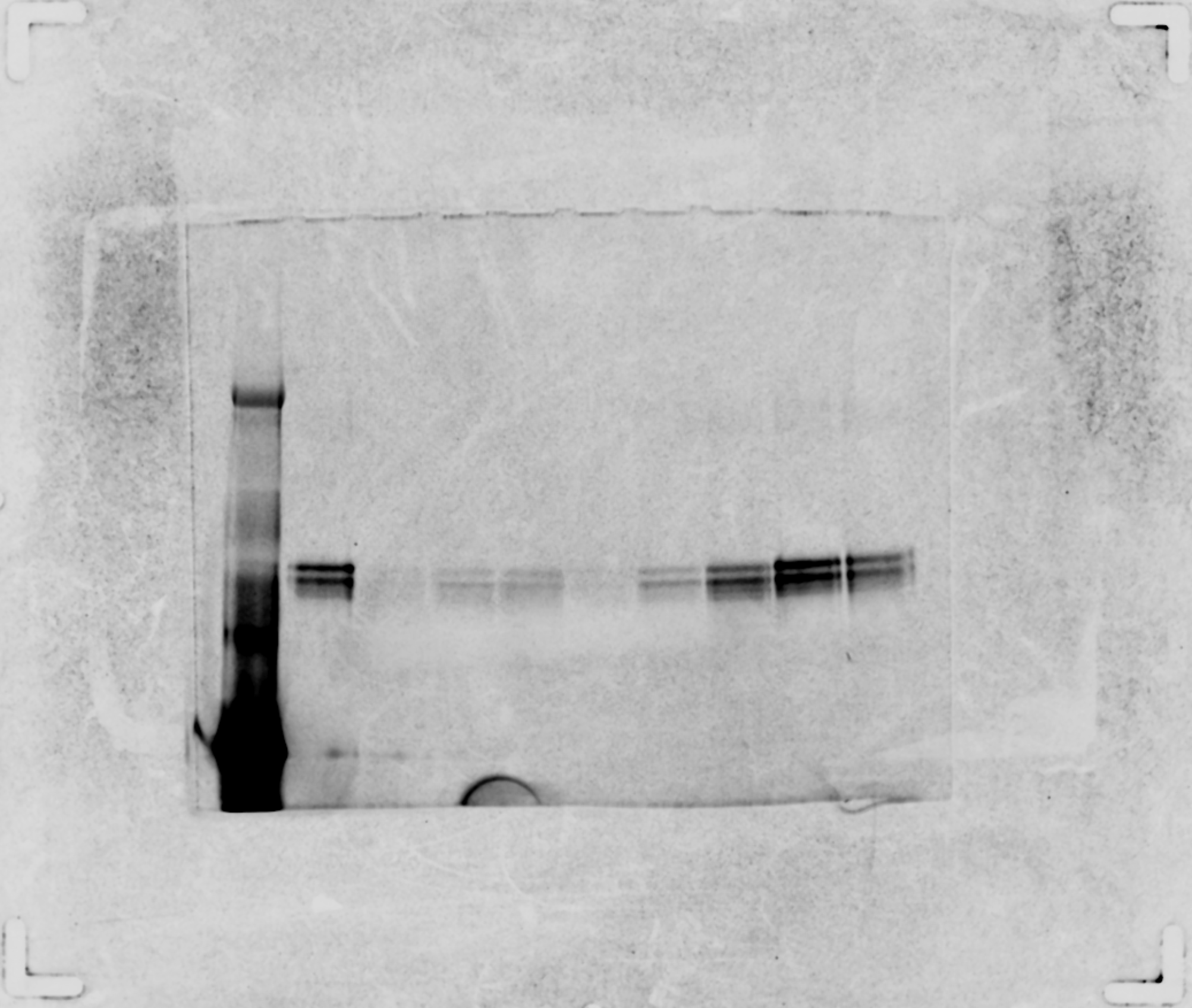

Supplement: Figure 6—source data 1. [file elife-85096-fig6-data1.zip › Figure6_sourcedata/6D_TAMRA.tif]

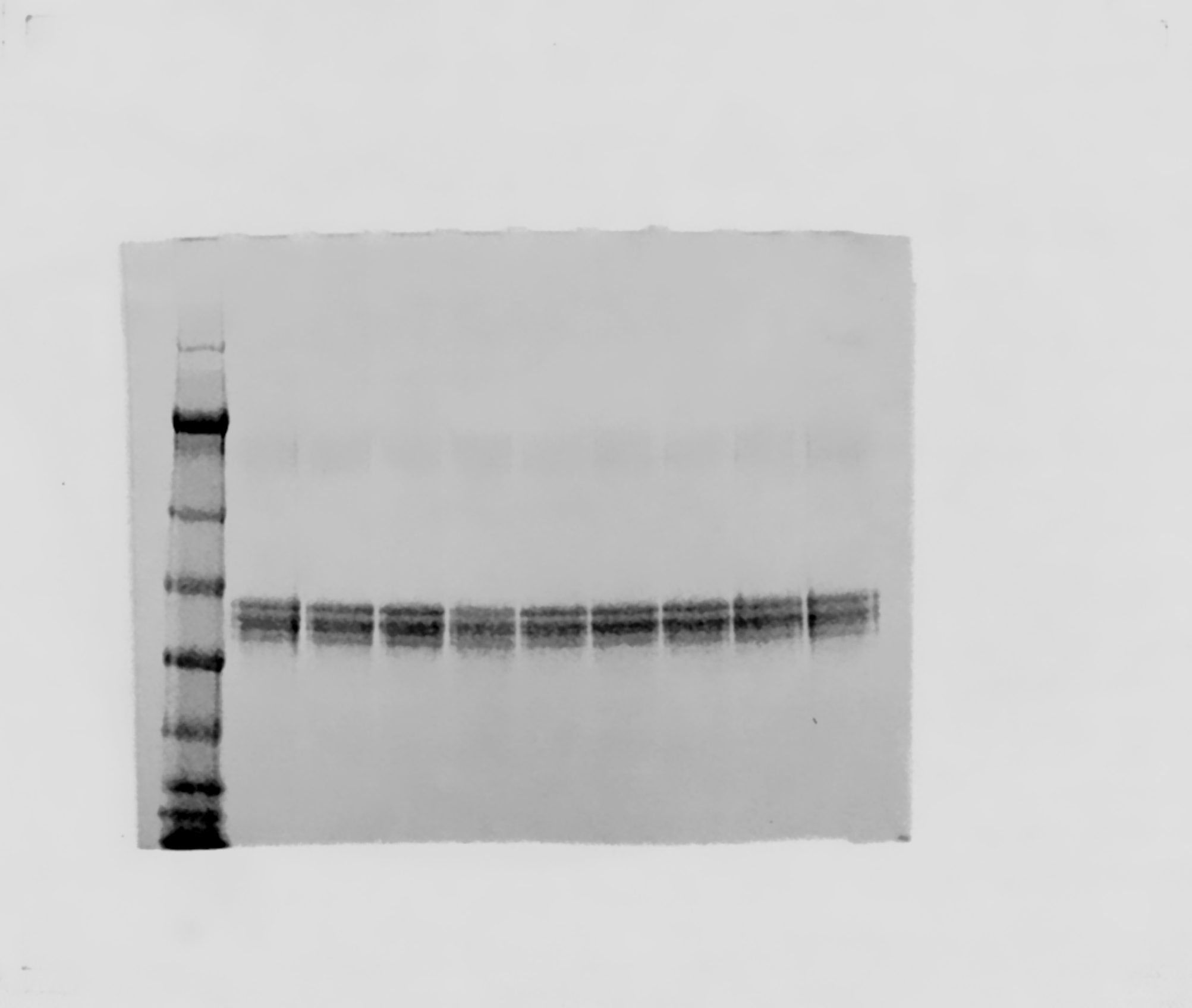

Supplement: Figure 6—source data 1. [file elife-85096-fig6-data1.zip › Figure6_sourcedata/6D_coomassie.tif]

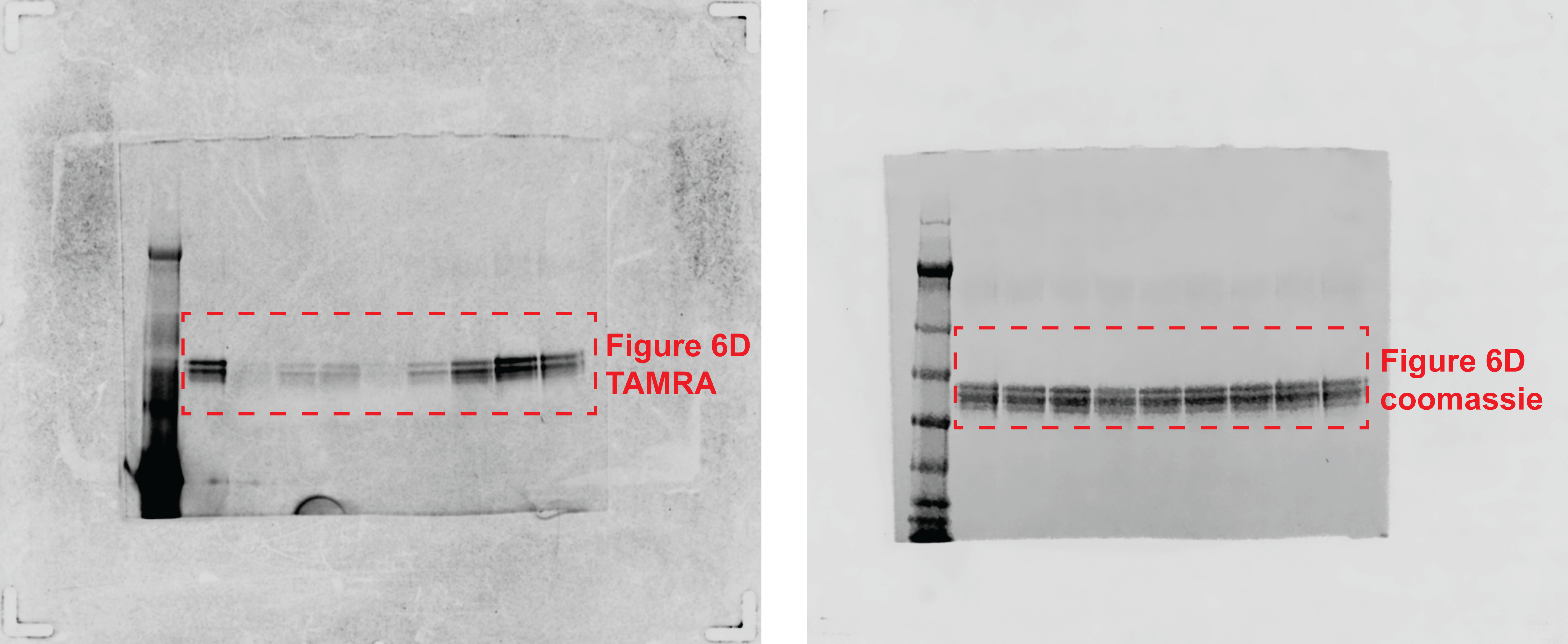

Supplement: Figure 6—source data 1. [file elife-85096-fig6-data1.zip › Figure6_sourcedata/Figure6_gelCroppingBounds.png]

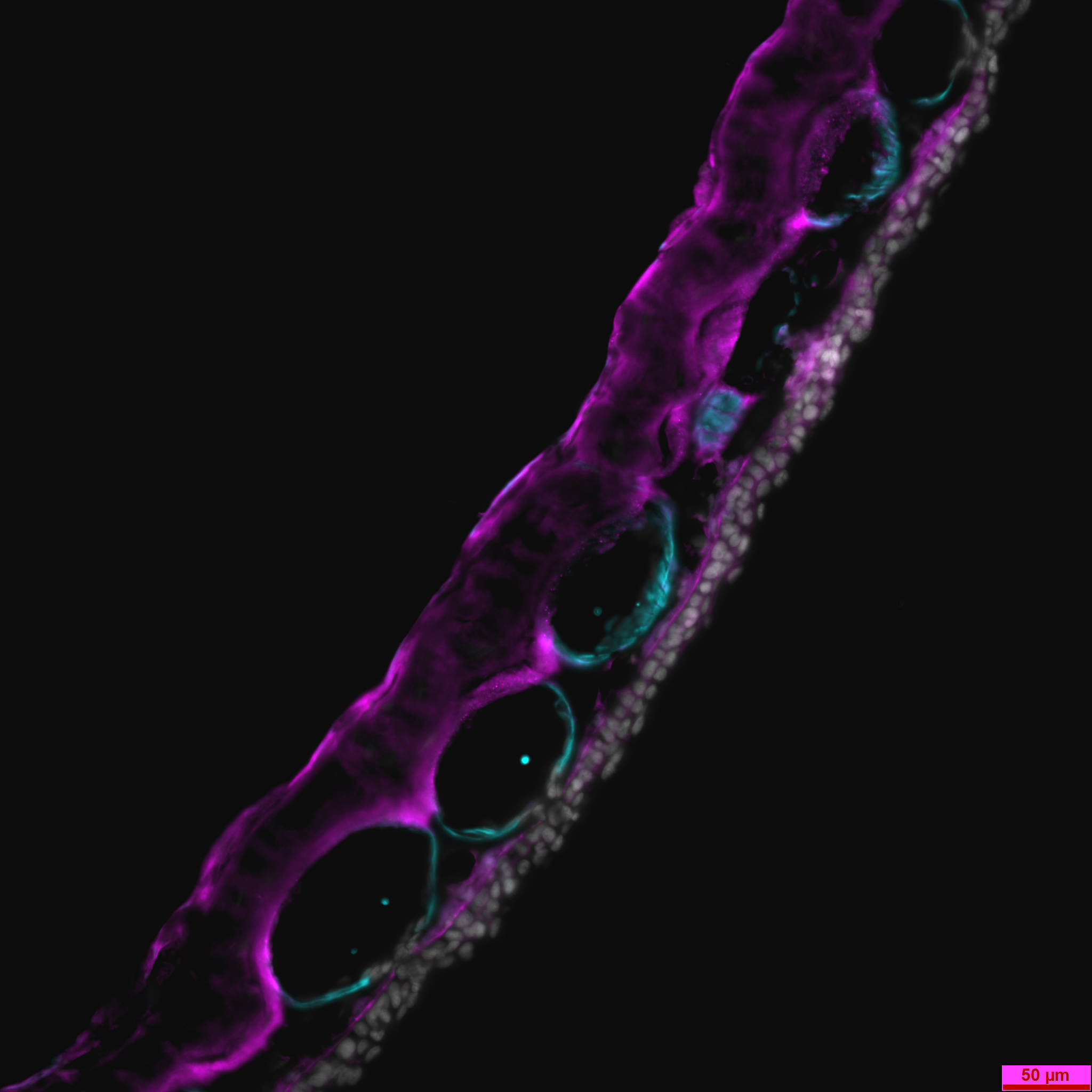

Supplement: Figure 6—source data 1. [file elife-85096-fig6-data1.zip › Figure6_sourcedata/6EF/6E/skin_Actin488_ABG568_20x_001.tif]

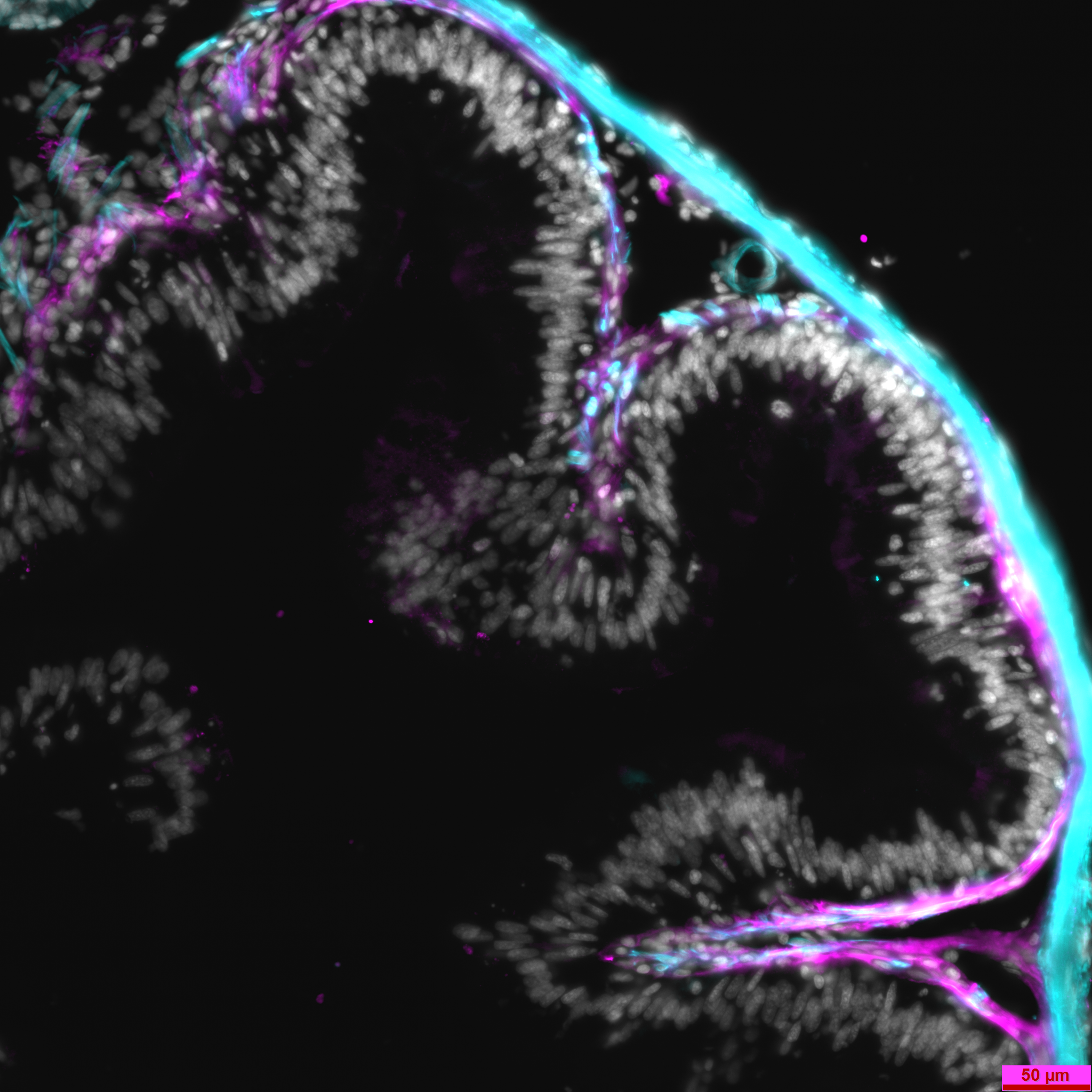

Supplement: Figure 6—source data 1. [file elife-85096-fig6-data1.zip › Figure6_sourcedata/6EF/6E/intestine_Actin488_ABG568_20x_004.tif]

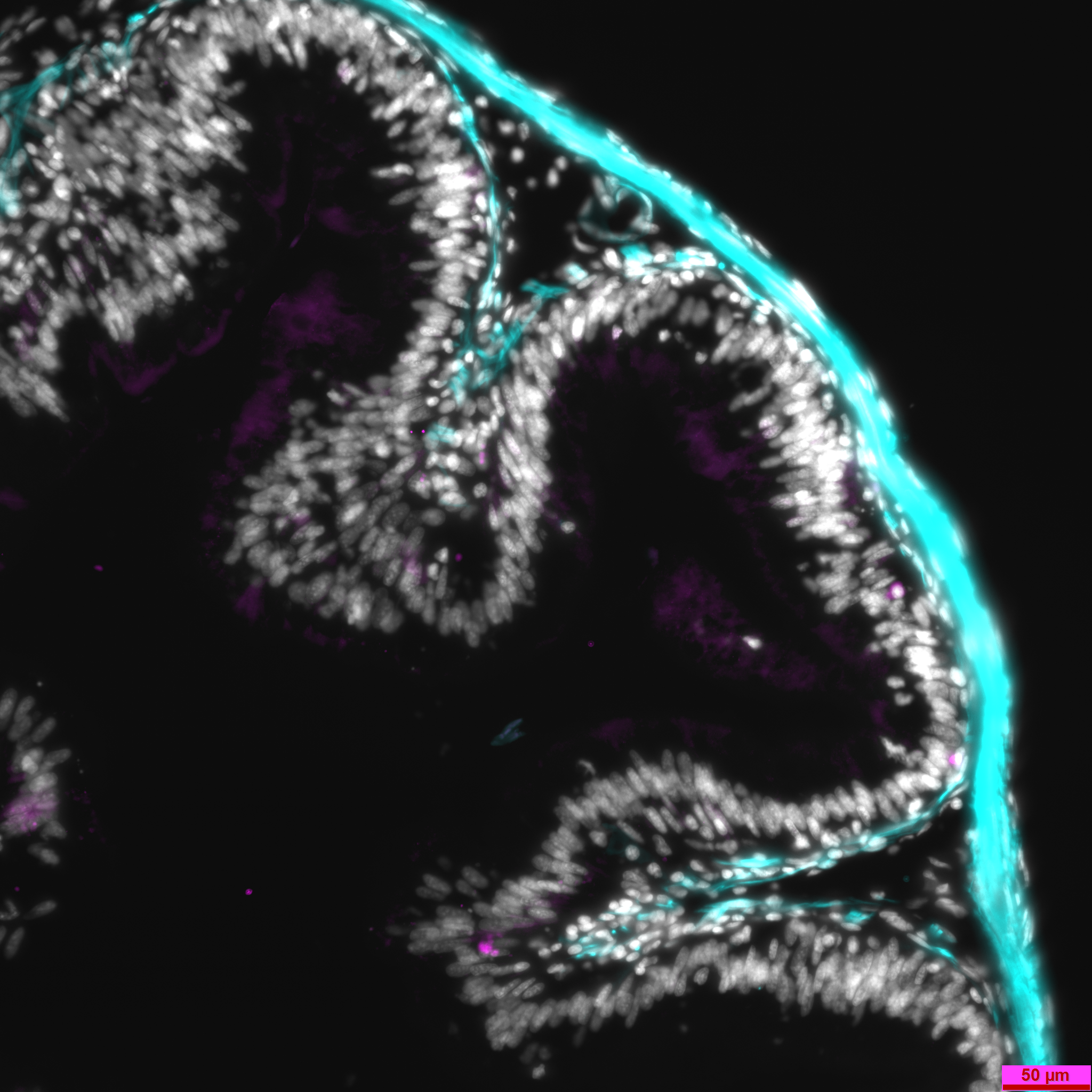

Supplement: Figure 6—source data 1. [file elife-85096-fig6-data1.zip › Figure6_sourcedata/6EF/6F/intestine_Actin488_ABG568_20x_protein5x_002.tif]

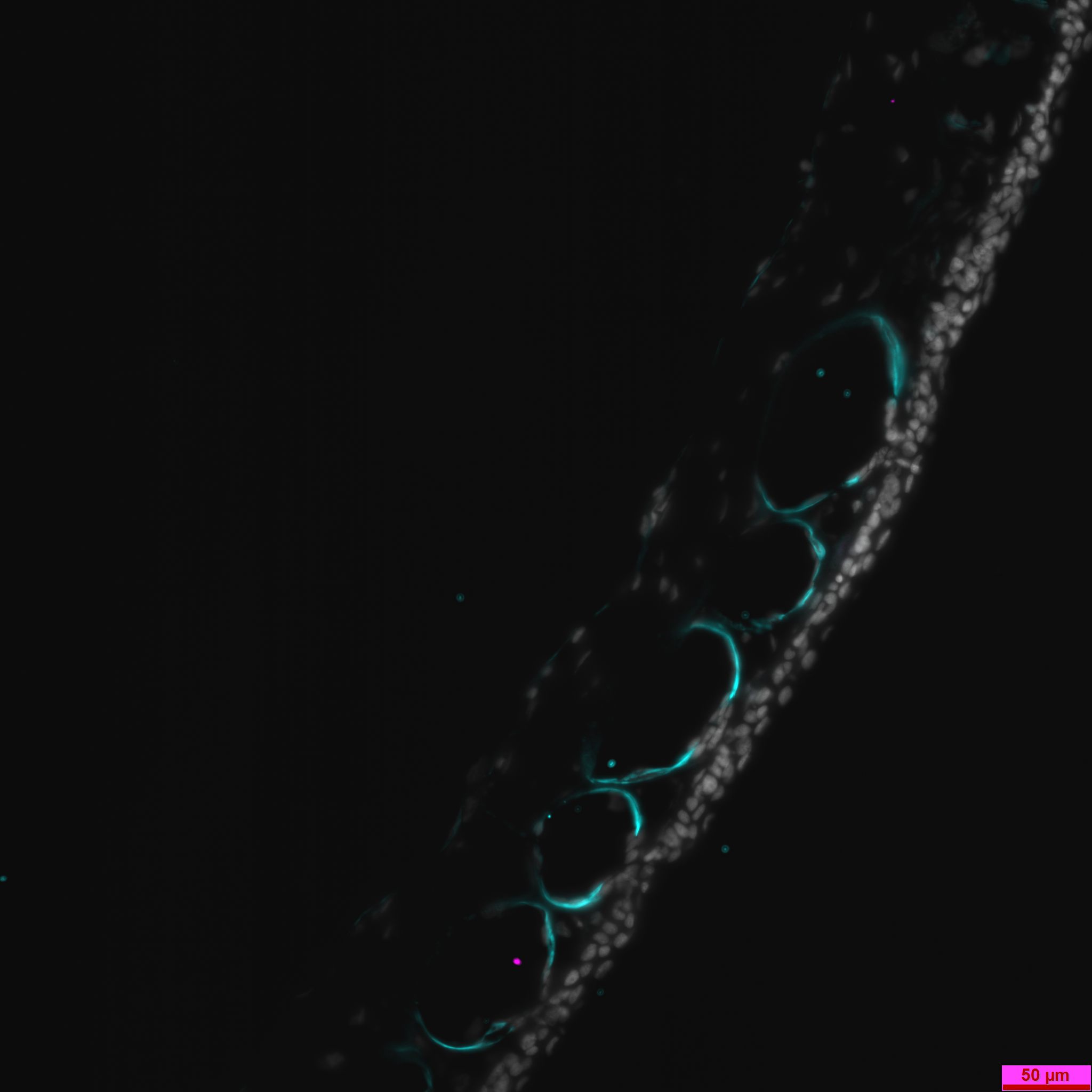

Supplement: Figure 6—source data 1. [file elife-85096-fig6-data1.zip › Figure6_sourcedata/6EF/6F/skin_Actin488_ABG568_20x_protein5x_001.tif]

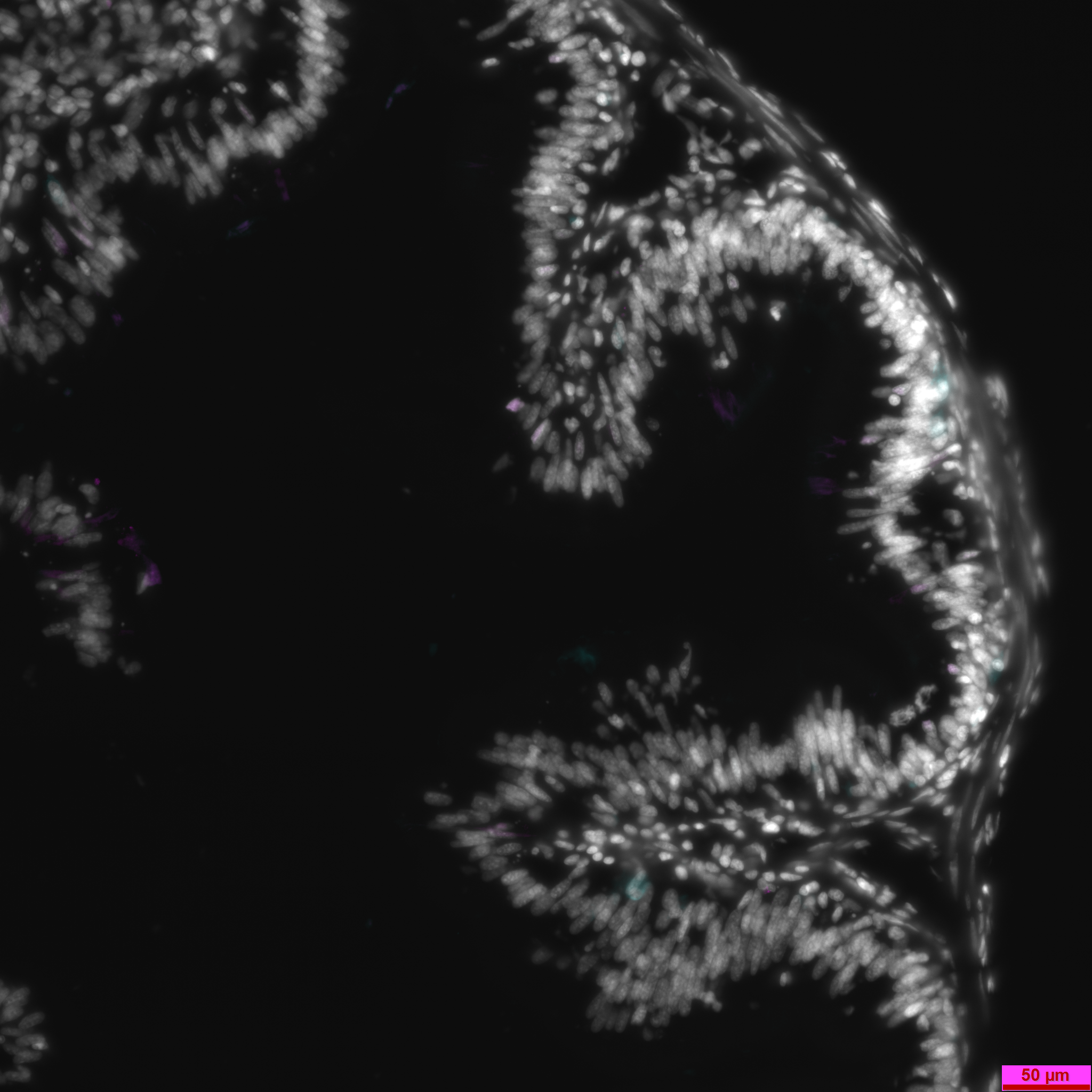

Supplement: Figure 6—figure supplement 2—source data 1. [file elife-85096-fig6-figsupp2-data1.zip › Figure6-fs2-sd1/intestine_Actin488_ABG568_20x_negative_003.tif]

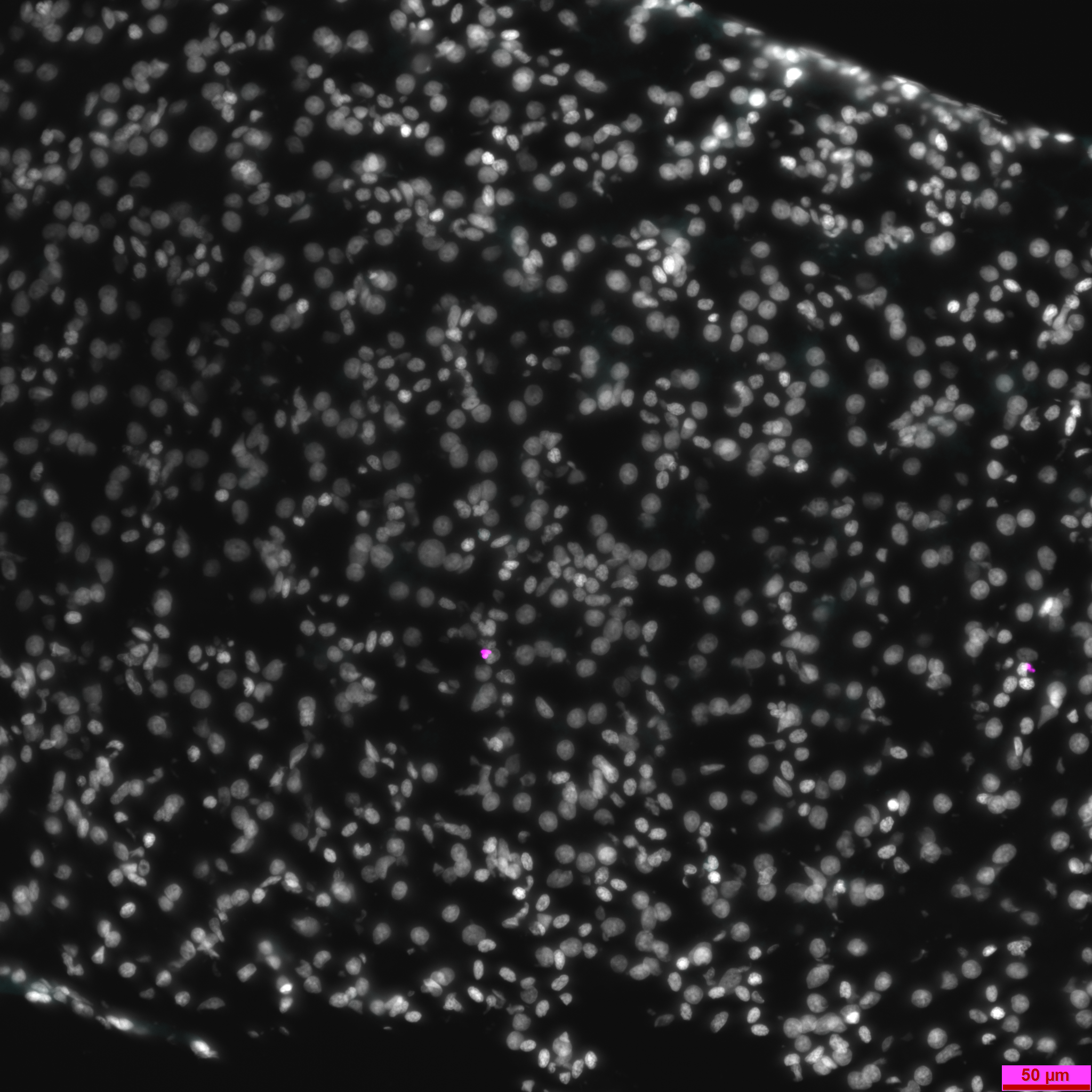

Supplement: Figure 6—figure supplement 2—source data 1. [file elife-85096-fig6-figsupp2-data1.zip › Figure6-fs2-sd1/liver_Actin488_ABG568_negative_20x_001.tif]

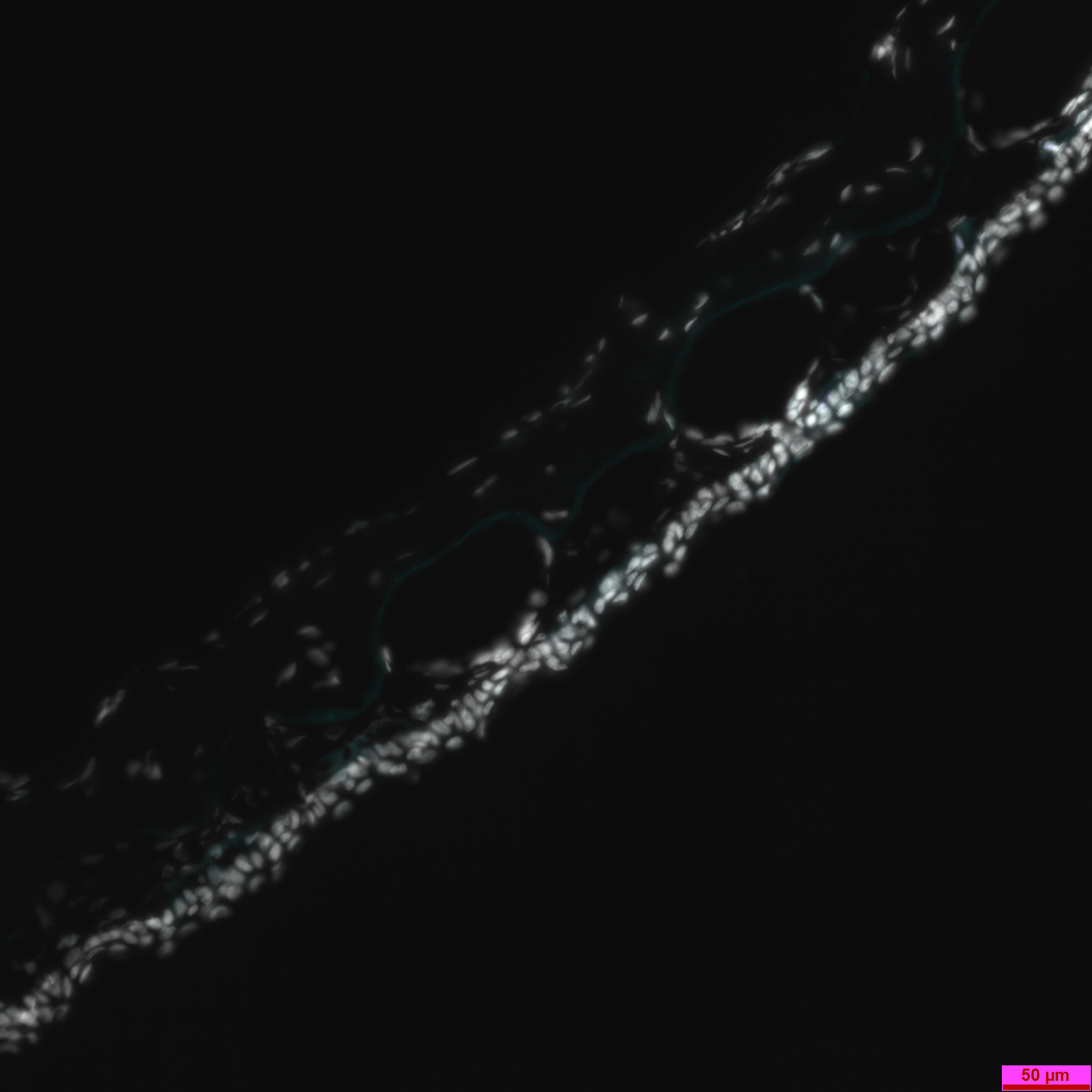

Supplement: Figure 6—figure supplement 2—source data 1. [file elife-85096-fig6-figsupp2-data1.zip › Figure6-fs2-sd1/skin_Actin488_ABG568_20x_negative_002.tif]

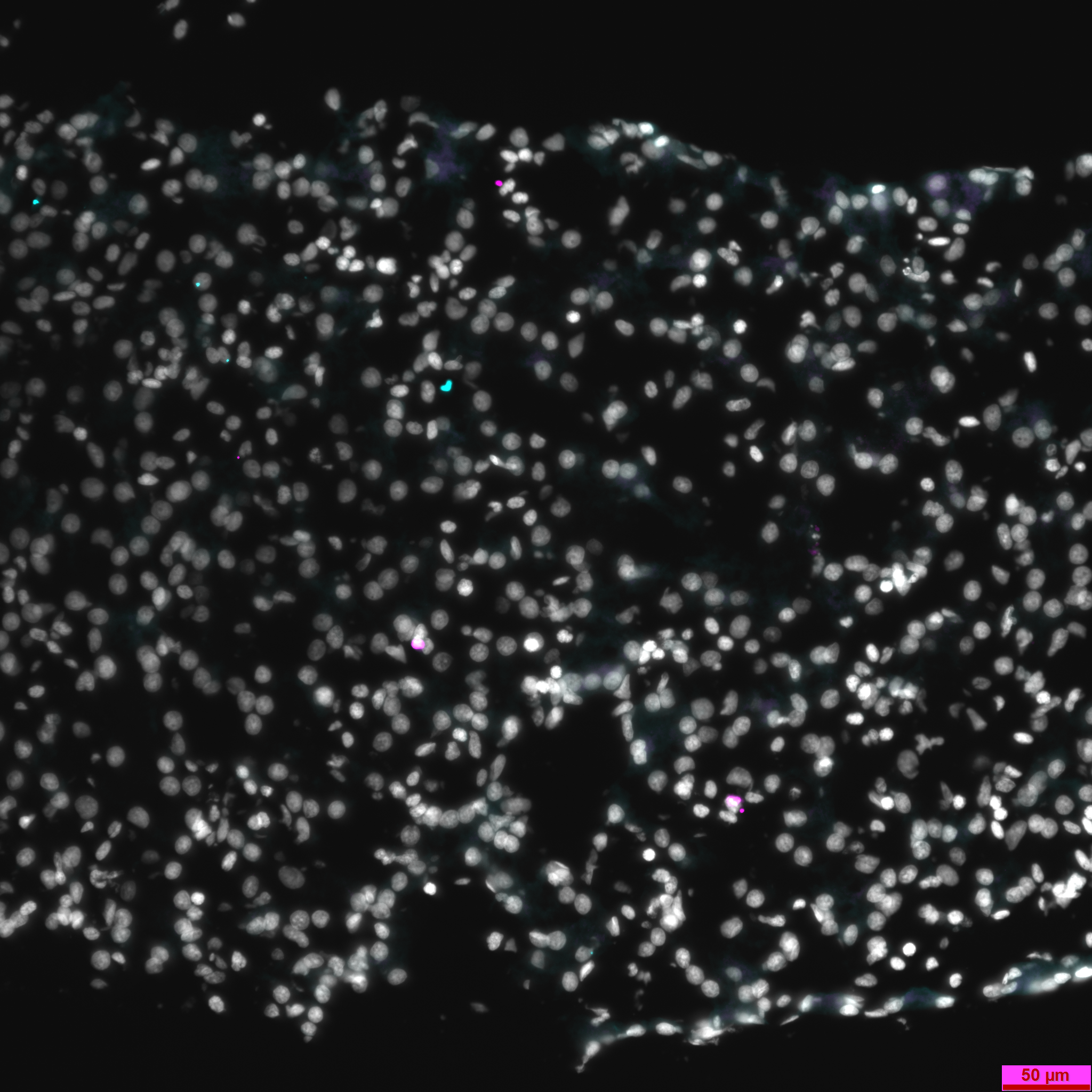

Supplement: Figure 6—figure supplement 2—source data 1. [file elife-85096-fig6-figsupp2-data1.zip › Figure6-fs2-sd1/liver_Actin488_ABG568_20x_protein5x_001.tiff]

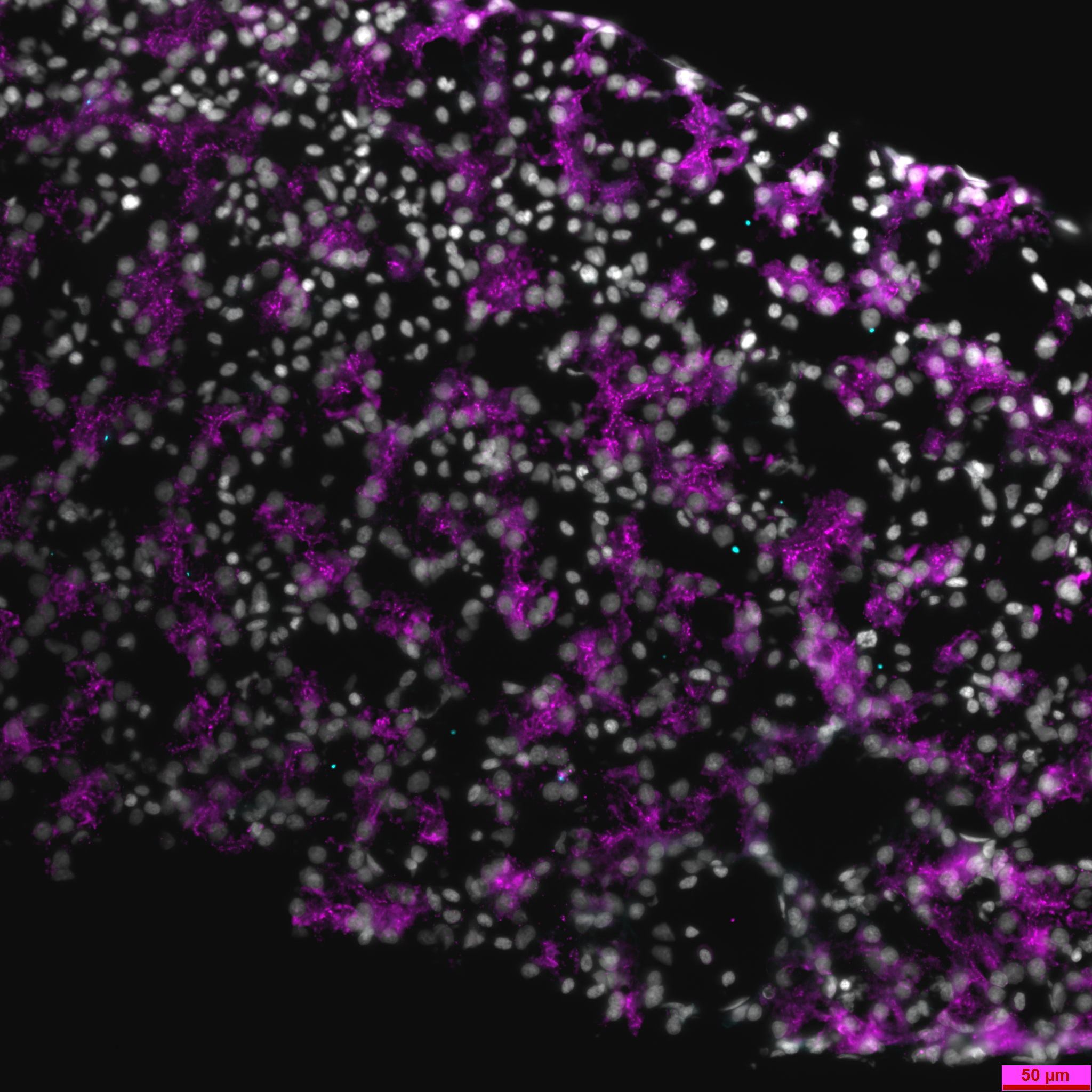

Supplement: Figure 6—figure supplement 2—source data 1. [file elife-85096-fig6-figsupp2-data1.zip › Figure6-fs2-sd1/liver_Actin488_ABG568_20x_001.tiff]

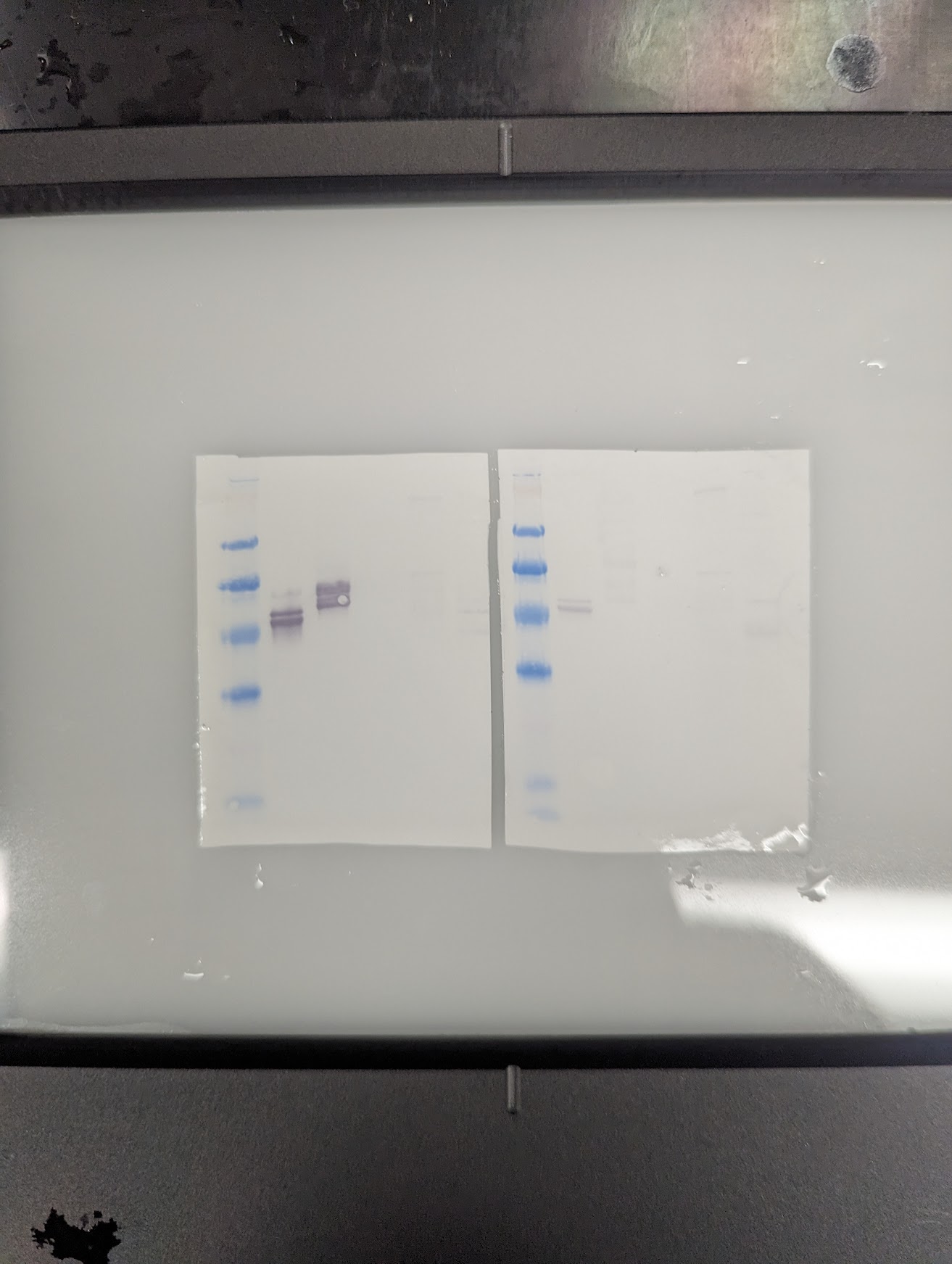

Supplement: Figure 6—figure supplement 3—source data 1. [file elife-85096-fig6-figsupp3-data1.zip › Figure6-fs3-sd1/S5B_HRPblot.jpeg]

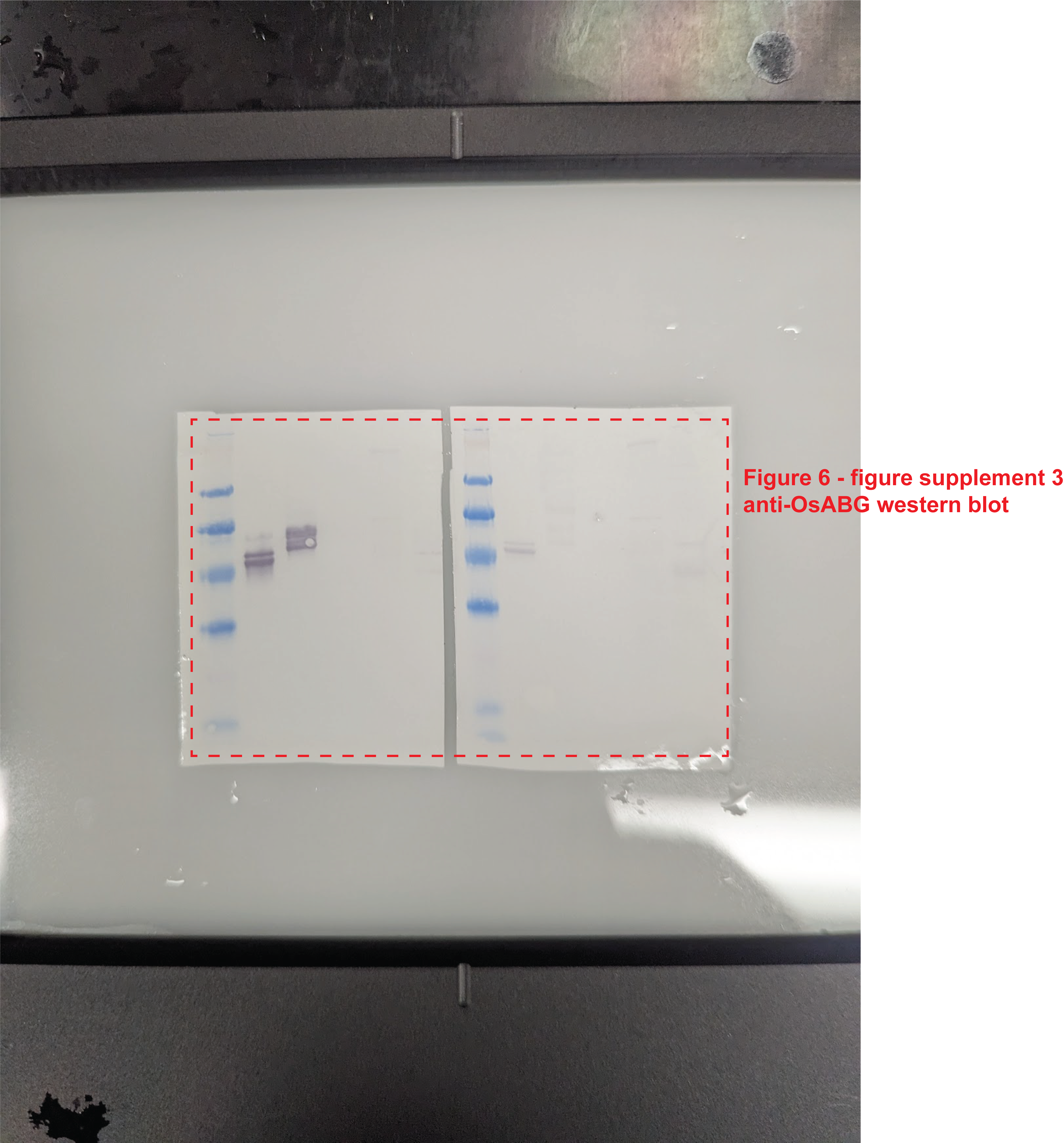

Supplement: Figure 6—figure supplement 3—source data 1. [file elife-85096-fig6-figsupp3-data1.zip › Figure6-fs3-sd1/Figure6_fs3_blotCroppingBounds.png]
